# Fast Cryomediated Dynamic Equilibrium Hydrolysates towards Grain Boundary-Enriched Platinum Scaffolds for Efficient Methanol Oxidation

**DOI:** 10.34133/2019/8174314

**Published:** 2019-10-13

**Authors:** Chao Zhang, Huajie Huang, Jianan Gu, Zhiguo Du, Bin Li, Songmei Li, Shubin Yang

**Affiliations:** ^1^Key Laboratory of Aerospace Advanced Materials and Performance of Ministry of Education, School of Materials Science and Engineering, Beihang University, 100191 Beijing, China; ^2^College of Mechanics and Materials, Hohai University, Nanjing 210098, China

## Abstract

Although platinum nanocrystals have been considered as potential electrocatalysts for methanol oxidation reaction (MOR) in fuel cells, the large-scale practical implementation has been stagnated by their limited abundance, easy poisoning, and low durability. Here, grain boundary-enriched platinum (GB-Pt) scaffolds are produced in large scale via facilely reducing fast cryomediated dynamic equilibrium hydrolysates of platinum salts. Such plentiful platinum grain boundaries are originated from the fast fusion of short platinum nanowires during reduction of the individually and homogeneously dispersed platinum intermediates. These grain boundaries can provide abundant active sites to efficiently catalyze MOR and meanwhile enable to oxidize the adsorbed poisonous CO during the electrocatalytic process. As a consequence, the as-synthesized GB-Pt scaffolds exhibit an impressively high mass activity of 1027.1 mA mg_Pt_^−1^ for MOR, much higher than that of commercial Pt/C (345.2 mA mg_Pt_^−1^), as well as good stability up to 5000 cycles. We are confident that this synthetic protocol can be further extended to synthesize various grain boundary-enriched metal scaffolds with broad applications in catalysis.

## 1. Introduction

Direct methanol fuel cells (DMFCs) have been demonstrated as one of the most promising power sources for electronic mobile devices and electric vehicles due to their ultrahigh energy densities and low pollution [1–3]. However, their wide applications have stagnated owing to the notoriously sluggish kinetics of methanol oxidation reaction (MOR) at the anode [4–8]. Thus, it is inevitable to develop high active precious metal electrocatalysts like platinum (Pt) to reduce the energy barriers of MOR [9–11]. From the perspective of Pt atomic efficiency [12], the precisely controlled synthesis of Pt nanocrystals such as Pt irregular nanoparticles [13, 14], nanowires [15–18], and nanorods [19] is highly desirable since they have inherent anisotropic morphologies with abundant low-coordinated surface atoms [20, 21], enabling to slow down the ripening process and increase the electrocatalytic activities for methanol oxidation.

Very recently, it is demonstrated that Pt nanocrystals with twin defects or dislocations have shown unique electrocatalytic behaviors for methanol oxidation, differentiating from coarsely gained grains, single crystals, or particles [21–23], since Pt atoms close to defects have decreased the number of neighbors in the first coordination shell, and are favorable to forcefully adsorb the reactants and catalyze related bond-breaking reactions during the catalytic process [24, 25]. Thus, the emergence of abundant defects, dislocations, or grain boundaries not only reduce the activation energy for methanol oxidation but also afford abundant catalytic sites for the oxidation of the adsorbed CO intermediate, significantly improving their stabilities [26, 27]. Hence, there is great interest in Pt nanocrystals with a high density of grain boundaries for electrocatalytic MOR [20, 26]. Unfortunately, to date, it remains a big challenge to synthesize grain boundary-enriched Pt nanocrystals via a facile and cost-efficient approach [28, 29].

Here, 3D grain boundary-enriched Pt scaffolds (3D GB-Pt scaffolds) are facilely produced via reducing fast cryogenic hydrolysates of ammonium hexachloroplatinate ((NH_4_)_2_PtCl_6_) aqueous solution. These grain boundaries are originated from the rapid coalescence of short Pt nanowires (~13.2 nm) during reduction of the individually and homogeneously dispersed platinum hydrolysates. These grain boundaries can provide abundant active sites to efficiently catalyze MOR and meanwhile enable to oxidize the adsorbed poisonous CO during the electrocatalytic process. Coupled to the 3D interconnected networks, both mass transport and electron transfer during MOR are fast in 3D GB-Pt scaffolds. As a consequence, the as-synthesized 3D GB-Pt scaffolds show an ultrahigh mass activity of 1027.1 mA mg_Pt_^−1^ for MOR, much higher than that of commercial Pt/C (345.2 mA mg_Pt_^−1^), and long-term durability up to 5000 cycles (only 9.8% loss of the initial activity).

## 2. Results

### 2.1. Preparation of 3D GB-Pt Scaffolds

**Figure 1:**
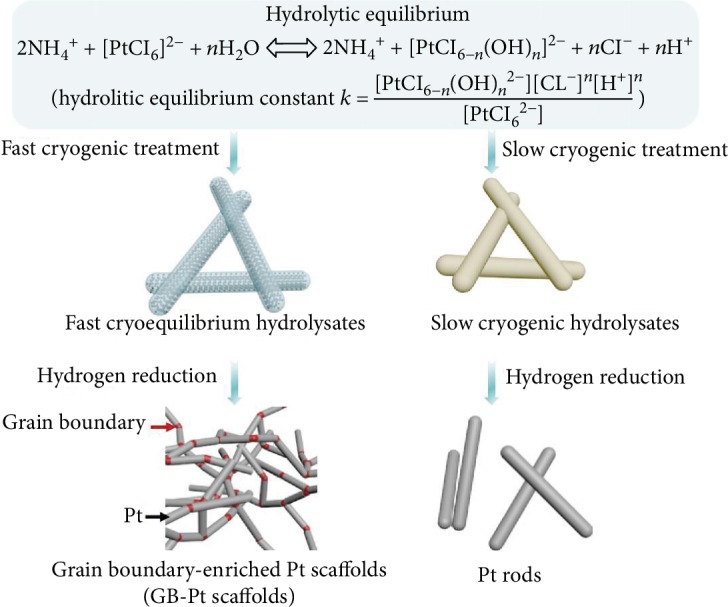
Schematic illustration of the synthesis of 3D GB-Pt scaffolds. The procedure for preparing 3D GB-Pt scaffolds involves two steps: (1) fast cryogenic treatment of the (NH_4_)_2_PtCl_6_ solution to produce fast cryogenic hydrolysates; (2) hydrogen reduction of the obtained fast cryogenic hydrolysates.

As illustrated in Figure 1 and [Supplementary-material supplementary-material-1], 3D GB-Pt scaffolds were synthesized through reduction of fast cryogenic hydrolysates of platinum salts in aqueous solution. Specifically, (NH_4_)_2_PtCl_6_ (5 mg) was dissolved in 10 ml deionized water, forming a pale-yellow solution. After standing for 10 h, the hydrolysis of (NH_4_)_2_PtCl_6_ reaches a dynamic equilibrium as follows [30, 31]:

(1) $$\begin{array}{c} 2{\text{NH}}_{4}^{+}+{\left[{\text{PtCl}}_{6}\right]}^{2-}+{n\text{H}}_{2}\text{O}⇋2{\text{NH}}_{4}^{+}+{\left[{\text{PtCl}}_{6-n}{\left(\text{OH}\right)}_{n}\right]}^{2-}+{n\text{Cl}}^{-}+{n\text{H}}^{+} \end{array}$$

where the hydrolysis equilibrium could be adjusted via tuning the pH values and the concentration of Cl^−^ during the hydrolysis process ([Supplementary-material supplementary-material-1]). After fast cryogenic treatment (the treatment temperature is -196°C), a yellow foam with equilibrium hydrated [PtCl_6−*n*_(OH)*_n_*]^2−^, NH_4_^+^, Cl^−^, and nonhydrolytic [PtCl_6_]^2−^ was formed. Then, the foam was reduced at 200°C under a mixed gas of H_2_ and Ar with a *v*/*v* ratio of 1 : 9 for 2 h, affording 3D GB-Pt scaffolds. In contrast, Pt rods were generated through reduction of the slow cryogenic hydrolysates, since hydrolytic equilibrium of (NH_4_)_2_PtCl_6_ went reversely back during the slow cryogenic process.

### 2.2. Effect of Fast Dynamic Equilibrium Hydrolysates to Guide 3D GB-Pt Scaffold Formation

**Figure 2:**
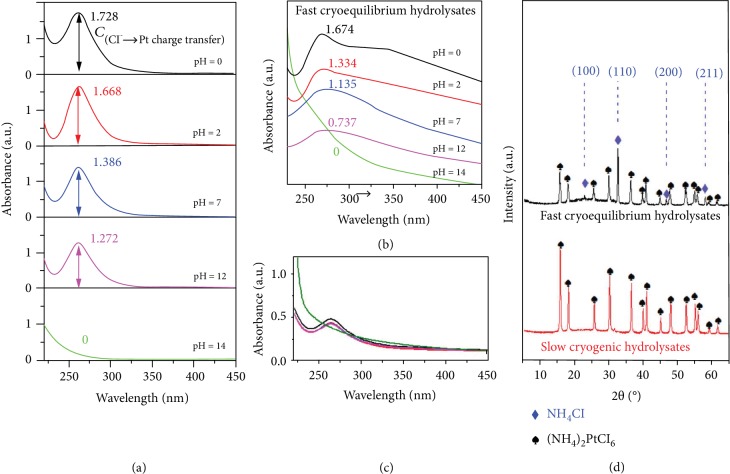
Detailed structural characterizations of fast and slow cryogenic hydrolysates. (a) UV-vis absorption spectra of (NH_4_)_2_PtCl_6_ aqueous solutions (0.1 mg ml^−1^) with different pH values (from 0 to 14), clearly indicating that the [PtCl_6_]^2-^ ion undergoes deep hydrolysis with increasing pH values. (b, c) UV-vis adsorption spectra of fast cryoequilibrium hydrolysates (b) and slow cryogenic hydrolysates (c) obtained from the (NH_4_)_2_PtCl_6_ solution with different pH values (from 0 to 14), showing that the hydrolysates at the different dynamic equilibrium states could be well maintained through fast cryogenic treatment. (d) XRD patterns of fast cryoequilibrium hydrolysates and slow cryogenic hydrolysates confirm the existence of NH_4_Cl in the obtained fast cryoequilibrium hydrolysates.

To identify the dynamic equilibrium hydrolysates of (NH_4_)_2_PtCl_6_ during our fast cryogenic treatment, UV-vis absorption measurement was conducted owing to its high sensitivity to ligand-to-metal (Cl^−^➜Pt) charge transfer of [PtCl_6_]^2−^ and [PtCl_6−*n*_(OH)*_n_*]^2−^ complexes [32, 33]. In principle, by adjusting the pH values of (NH_4_)_2_PtCl_6_ solution from 0 to 12, the different dynamic equilibriums could be achieved, as demonstrated by their UV-vis absorption spectra with different absorbance intensities at ~262 nm (Figure 2(a)), which were originated from the charge transfer involving orbitals with Cl ligand *π*-character [30, 33]. Notably, while adjusting the pH value to 14, all the (NH_4_)_2_PtCl_6_ was conversed to [Pt(OH)_6_]^2−^, Cl^−^, and NH_4_^+^, where the hydrolytic equilibrium was completely broken [34]. Through fast cryogenic treatment, the hydrolysates at the different dynamic equilibrium states could be well maintained. This could be clearly demonstrated via their UV-vis absorption spectra (Figure 2(b)). On the contrary, through slow cryogenic treatment (the treatment temperature is -5°C), all the adsorption bands of the hydrolysates at the different dynamic equilibrium states showed the similar absorption intensities (Figure 2(c)). It is suggested that the different levels of hydrolysate [PtCl_6−*n*_(OH)*_n_*]^2−^ were recrystallized to (NH_4_)_2_PtCl_6_ during our slow cryogenic process.

**Figure 3:**
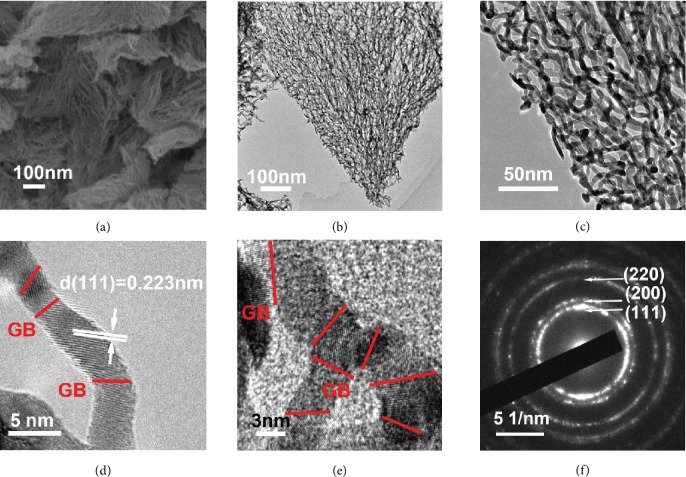
Morphological and structural characterizations of 3D GB-Pt scaffolds. (a) FESEM image of 3D GB-Pt scaffolds. (b, c) TEM images of 3D GB-Pt scaffolds with different magnifications. (d, e) HRTEM images of 3D GB-Pt scaffolds, exhibiting clear grain boundaries and *d*-spacing values of 0.223 nm (Pt (111) lattices). The grain boundaries are marked with red lines in (d) and (e). (f) SAED patterns of 3D GB-Pt scaffolds.

To further evaluate the crystalline phases of fast cryogenic intermediates, X-ray diffraction (XRD) measurement was conducted and is shown in Figure 2(d). Interestingly, in the case of fast cryogenic intermediates, except for the characterization peaks of (NH_4_)_2_PtCl_6_, there are four additional XRD peaks detected at 22.9°, 32.6°, 46.9°, and 58.3° (marked with blue diamonds in Figure 2(d)), indexed to (100), (110), (200), and (211) planes of NH_4_Cl (JCPDS no. 72-2378), respectively. The typical TEM ([Supplementary-material supplementary-material-1]) and elemental mapping images (shown in [Supplementary-material supplementary-material-1]) reveal that the product NH_4_Cl behaves like a matrix to accommodate the homogeneous dispersion of the fast cryogenic Pt-containing intermediates with sizes of 3-5 nm. Hence, through initial hydrogen reduction treatment, numerous individual Pt nanocrystalline structures could be produced, derived from Pt-containing intermediates. Meanwhile, NH_4_Cl would be gradually decomposed and leave gaps between the Pt nanocrystalline structures. With further increase of the reduction time, such gaps allow the infusion between adjacent Pt nanocrystalline structures, generating grain boundary-enriched Pt scaffolds as demonstrated in Figure 3 and Figures [Supplementary-material supplementary-material-1] and [Supplementary-material supplementary-material-1]. The typical TEM images (Figure 3(b)–3(e) and [Supplementary-material supplementary-material-1]) clearly disclose that these scaffolds are constructed from short Pt crystalline wires with average lengths of ~13 nm and diameters of ~4.2 nm. HRTEM images (Figures 3(d) and 3(e) and [Supplementary-material supplementary-material-1]) show clearly the grain boundaries between two short Pt wires and an obvious interlayer spacing of 0.223 nm, corresponding to *d*(111) of Pt [35, 36]. In contrast, different levels of hydrolysate [PtCl_6−*n*_(OH)*_n_*]^2−^ and Cl^−^ were recrystallized to single-crystalline (NH_4_)_2_PtCl_6_ during the slow cryogenic treatment, without the detection of equilibrium products of the hydrolysis (Figure 2(d) and [Supplementary-material supplementary-material-1]). After hydrogen reduction treatments of slow cryogenic intermediates, only Pt rods were obtained (Figures [Supplementary-material supplementary-material-1] and [Supplementary-material supplementary-material-1]). This may be ascribed to the low level of NH_4_Cl in the slow cryogenic intermediates, which are unable to prevent the fast growth of big Pt crystalline structures. Moreover, pH values of the (NH_4_)_2_PtCl_6_ solution also affect the formation of 3D GB-Pt scaffolds. As shown in Figures [Supplementary-material supplementary-material-1] and [Supplementary-material supplementary-material-1], while the pH value is 2 and 7, the resultant GB-Pt scaffolds show interconnected 3D networks. As the pH value is 0, all the nanowires or particles are strongly aggregated, owing to the less amount of the hydrolysis product NH_4_Cl, which cannot efficiently prevent the aggregation during reduction process. In contrast, as the pH value arrives to 12 and 14, all the Pt nanocrystalline structures are separated, without formation of good networks. This should be ascribed to the excessive hydrolysis product NH_4_Cl after fast cryogenic treatment, which prevents the infusion between the formed Pt nanocrystalline structures during the reduction process. These results manifest that the appropriate amount of NH_4_Cl plays a key role to the formation of 3D grain boundary-enriched Pt scaffolds. To further confirm this hypothesis, two chloroplatinates without ammonium (Na_2_PtCl_6_ and K_2_PtCl_6_) were selected to substitute the platinum precursor (NH_4_)_2_PtCl_6_ during our fabrication processes. As shown in [Supplementary-material supplementary-material-1], only Pt nanoparticles were generated after reduction of the fast cryoequilibrium hydrolysates of K_2_PtCl_6_. If we deliberately added an amount of NH_4_Cl into the K_2_PtCl_6_ system, 3D Pt scaffolds could be formed again ([Supplementary-material supplementary-material-1] and [Supplementary-material supplementary-material-1]). This phenomenon appears again as Na_2_PtCl_6_ was used as the Pt precursor (Figures [Supplementary-material supplementary-material-1] and [Supplementary-material supplementary-material-1]). Therefore, the presence of a moderate amount of NH_4_Cl is the essence of the production of 3D grain boundary-enriched Pt scaffolds. In this manner, various grain boundary-enriched metal (e.g., Pd) scaffolds could be fabricated via our fast cryogenic treatment with the presence of a NH_4_Cl additive, and the detailed characterizations of 3D GB-Pd scaffolds are shown in the Supplementary Materials ([Supplementary-material supplementary-material-1]).

It should be noted that the grain boundary density of the sample can be well controlled through tuning of the hydrolysis equilibrium by adjusting the concentrations of (NH_4_)_2_PtCl_6_ solution before fast cryogenic treatment. As shown in [Supplementary-material supplementary-material-1], a higher concentration of (NH_4_)_2_PtCl_6_ leads to an increase of the length of Pt wires. Based on the TEM analysis, we quantitatively measured the average Pt grain length of the GB-Pt scaffolds. It is shown that the average grain length of GB-Pt scaffolds-5 is only 13.2 nm (the grain boundary density of 75.9 *μ*m^−1^), which is the highest grain boundary density among all the GB-Pt scaffolds samples. (For details, see the Supplementary Materials.) In this manner, the grain boundary density of 3D GB-Pd scaffolds was 64.1 *μ*m^−1^ ([Supplementary-material supplementary-material-1]).

### 2.3. Structural and Compositional Analysis of 3D GB-Pt Scaffolds

**Figure 4:**
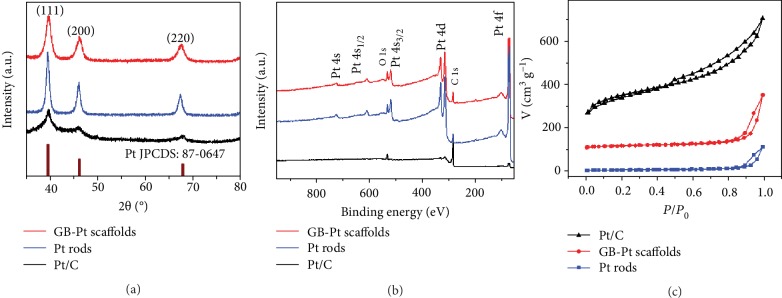
Structural characterizations of 3D GB-Pt scaffolds, Pt rods, and commercial Pt/C. (a) XRD patterns demonstrating that all peaks can be indexed to fcc Pt, (b) XPS spectra demonstrating that no other impurity elements were observed, and (c) nitrogen adsorption/desorption isotherm of 3D GB-Pt scaffolds, Pt rods, and Pt/C.

To gain further insight into the crystalline properties of 3D GB-Pt scaffolds, XRD, XPS, and nitrogen adsorption/desorption isotherm were carried out. As revealed in Figure 4(a) and Figures [Supplementary-material supplementary-material-1] and [Supplementary-material supplementary-material-1], in the case of 3D GB-Pt scaffolds, there are three prominent XRD peaks at 39.8°, 46.2°, and 67.7°, corresponding to the (111), (200), and (220) planes of face-centered-cubic (fcc) platinum (JCPDS no. 87-0647) [37], respectively, in good accordance with the above HRTEM and SAED analyses. The XPS survey demonstrates that there are only two main species of Pt and O detected in our 3D GB-Pt scaffolds, without other impurities (Figure 4(b) and Figures [Supplementary-material supplementary-material-1] and [Supplementary-material supplementary-material-1]). N_2_ adsorption-desorption data indicates a high specific surface area of 50.6 m^2^ g^−1^, much higher than that of the Pt rods (21.1 m^2^ g^−1^) sample (Figure 4(c)).

### 2.4. Electrocatalytic Properties of 3D GB-Pt Scaffolds for Methanol Oxidation

**Figure 5:**
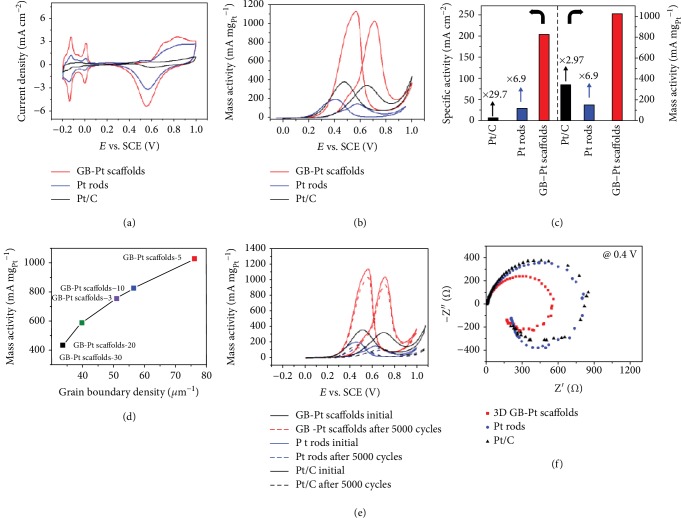
Electrocatalytic activities of 3D GB-Pt scaffolds for MOR. (a) Cyclic voltammograms (CVs) tested in 0.5 M H_2_SO_4_ electrolyte at 10 mV s^−1^. (b) CVs tested in 0.5 M H_2_SO_4_ and 1 M methanol electrolyte with 20 mV s^−1^. (c) Comparative Pt mass activity and specific activity of 3D GB-Pt scaffolds, Pt rods, and Pt/C electrocatalysts. (d) Relationship between grain boundary density and MOR mass activity of 3D GB-Pt scaffolds, showing that the electrocatalytic activities are linearly proportional to the densities of grain boundaries. (e) CVs of 3D GB-Pt scaffolds, Pt rods, and Pt/C before and after 5000 CV cycles, showing an excellent durability of the 3D GB-Pt scaffolds. (f) Nyquist plots of 3D GB-Pt scaffolds, Pt rods, and Pt/C electrocatalysts for methanol oxidation at 0.4 V.

The electrocatalytic activities of 3D GB-Pt scaffolds for MOR were investigated directly via cyclic voltammograms (CVs) in an electrolyte of 0.5 M H_2_SO_4_ and 1 M methanol. As displayed in Figure 5(a) and [Supplementary-material supplementary-material-1], a high electrochemically active surface area (ECSA) value of 74.8 m^2^ g_Pt_^−1^ is obtained in the case of GB-Pt scaffolds, much higher than in Pt rods (26.9 m^2^ g_Pt_^−1^) and Pt/C (35.9 m^2^ g_Pt_^−1^) ([Supplementary-material supplementary-material-1]). Accurately, as shown in Figures 5(b) and 5(c), an ultrahigh peak with current density of 203.8 mA cm^−2^ is achieved, corresponding to 1027.1 mA mg_Pt_^−1^ for 3D GB-Pt scaffolds in mass activity, much higher than that of the Pt/C sample (345.2 mA mg_Pt_^−1^) ([Supplementary-material supplementary-material-1]). Moreover, the onset potential of 3D GB-Pt scaffolds is only 217 mV, much lower than those of Pt rods (257 mV) and the Pt/C catalyst (356 mV). Associated with the above TEM and HRTEM analyses, such high electrocatalytic activities of 3D GB-Pt scaffolds should be attributed to the large presence of grain boundaries of Pt. This can be further demonstrated by the different 3D GB-Pt scaffolds with tunable intensities of grain boundaries, in which their activities are linearly proportional to the densities of grain boundaries of Pt (Figure 5(d)). The electrocatalytic activity of 3D GB-Pt scaffolds obtained at different pH values was also systematically investigated ([Supplementary-material supplementary-material-1]). Among them, 3D GB-Pt scaffolds-pH(7) exhibit a very high value of 1027.1 mA mg_Pt_^−1^, much higher than other samples (368.9, 586.9, 485.6, and 166.6 mA mg_Pt_^−1^ for 3D GB-Pt scaffolds-pH(0), (2), (12), and (14), respectively).

To further investigate the electrocatalytic stability of 3D GB-Pt scaffolds for MOR, we conducted a stability test in 0.5 M H_2_SO_4_ and 1 M methanol. Remarkably, even after 5000 cycles, there is only 9.8% loss of the initial activity for 3D GB-Pt scaffolds (Figure 5(e)). This value is much lower than that of the Pt/C catalyst (66.5% loss of the activity), clearly confirming the excellent durability of 3D GB-Pt scaffolds for electrocatalytic MOR. To gain insight into the reason of the excellent durability performance of 3D GB-Pt scaffolds, CO-stripping measurement was conducted [38]. As demonstrated in [Supplementary-material supplementary-material-1], the peak potential of 3D GB-Pt scaffolds is only 0.54 V (vs. SCE), much lower than that of Pt/C (0.61 V vs. SCE), suggesting that 3D GB-Pt scaffolds have an enhanced antipoisoning (CO) property [39]. This should ascribe to the presence of large Pt grain boundaries that can efficiently oxidize the adsorbed CO_ads_ [40]. Furthermore, after durability measurement, both the configurations and grain boundaries of 3D GB-Pt scaffolds were well preserved ([Supplementary-material supplementary-material-1]). The electrochemical impedance spectroscopy (EIS) of the 3D GB-Pt scaffolds, Pt rods, and Pt/C electrocatalysts was carried out to investigate the kinetics of methanol oxidation. As shown in Figures [Supplementary-material supplementary-material-1] and [Supplementary-material supplementary-material-1], typical methanol oxidation behaviors catalyzed by the Pt-based catalyst are observed at a series of potentials from 0.1 to 1.0 V [11]. Among them, 3D GB-Pt scaffolds have a much smaller semicircle diameter than Pt rods and Pt/C electrocatalysts at 0.4 V (Figure 5(f)), demonstrating the high methanol oxidation rate of 3D GB-Pt scaffolds [41], in accordance with the electrocatalytic MOR results (Figure 5(b)). In addition, 3D GB-Pd scaffolds also exhibit remarkably high activity of 1117.9 mA mg_Pd_^−1^ ([Supplementary-material supplementary-material-1]) and good stability (9.0% loss of the initial activity after 5000 CV cycles) towards MOR ([Supplementary-material supplementary-material-1]).

## 3. Discussion

In summary, grain boundary-enriched platinum scaffolds were produced in large scale via simply reducing fast cryomediated dynamic equilibrium hydrolysates of (NH_4_)_2_PtCl_6_. Such plentiful platinum grain boundaries are ascribed to the fast fusion of short platinum nanowires during reduction of the individually and homogeneously dispersed platinum-containing intermediates in the NH_4_Cl matrix, which is the essence of the production of grain boundary-enriched Pt scaffolds. These grain boundaries can provide abundant active sites to efficiently catalyze methanol oxidation, with an ultrahigh mass activity of 1027.1 mA mg_Pt_^−1^ for MOR, much higher than that of commercial Pt/C (345.2 mA mg_Pt_^−1^). Moreover, the mass activities of 3D GB-Pt scaffolds are linearly proportional to the densities of grain boundaries of Pt. And these grain boundaries enable oxidization of the adsorbed poisonous CO during the electrocatalytic process, leading to a long stability up to 5000 cycles. We are confident that such a simple synthetic protocol can be extended to produce various grain boundary-enriched metal scaffolds with broad applications for catalysis and sensors.

## 4. Materials and Methods

### 4.1. Materials

Ammonium hexachloroplatinate ((NH_4_)_2_PtCl_6_), potassium hexachloroplatinate (K_2_PtCl_6_), sodium hexachloroplatinate (Na_2_PtCl_6_), and ammonium hexachloropalladate ((NH_4_)_2_PdCl_6_) were purchased from Alfa Aesar. Ammonium chloride and sodium chloride were purchased from Beijing Innochem Technology Co., Ltd.

### 4.2. Synthesis of 3D GB-Pt Scaffolds

For the synthesis of 3D GB-Pt scaffolds, a certain amount of (NH_4_)_2_PtCl_6_ was initially added in DI water (10 ml) to form a pale-yellow solution. The solution was stirred for 60 min and then frozen at -196°C in liquid nitrogen for 30 min; after that, the obtained frozen product was further dried under vacuum conditions at -60°C for 48 h, and a three-dimensional yellow foam was obtained. The foam was further reduced at 200°C for 2 h under 10% H_2_/Ar gas, generating 3D GB-Pt scaffolds. 3D GB-Pt scaffolds-*X* were fabricated, where *X* represents the amount of (NH_4_)_2_PtCl_6_ in the 10 ml solution before fast cryogenic treatment. 3D GB-Pt scaffolds-pH(*Y*) were also fabricated, where *Y* represents the pH value of the (NH_4_)_2_PtCl_6_ solution before fast cryogenic treatment.

### 4.3. Synthesis of Pt Rods

The synthetic procedure of Pt rods was similar to that of 3D GB-Pt scaffolds except that the fast cryogenic treatment was replaced by slow cryogenic treatment (the freezing temperature changed form -196°C to -5°C).

### 4.4. Synthesis of 3D GB-Pd Scaffolds

The synthetic procedure of 3D GB-Pd scaffolds was similar to that of 3D GB-Pt scaffolds except that the (NH_4_)_2_PtCl_6_ was replaced by (NH_4_)_2_PdCl_6_, and 5 mg of NH_4_Cl should added into the (NH_4_)_2_PdCl_6_ solution before fast cryogenic treatment.

### 4.5. Characterization Methods

Nanostructures and morphologies of all the samples were carried out through FESEM (JEOL-7500) and HRTEM (JEOL, NEM-2100F). Nitrogen sorption isotherms and BET surface area were measured with Quadrasorb at 77 K. The powder X-ray diffraction patterns were conducted by a Rigaku D/max2500PC diffractometer with a Cu K*α* radiation over the range 35-80°. XPS data and chemical bonding nature of Pt and O elements were acquired using Thermo Scientific ESCALAB 250Xi X-ray photoelectron spectroscopy (XPS).

### 4.6. Electrocatalytic Measurements

Electrocatalytic properties of our samples were investigated by using an AutoLab workstation in a three-electrode setup cell. Typically, 3 mg of sample and 80 *μ*l of 5 wt% Nafion solution was dispersed in 1 ml of 4 : 1 *v*/*v* deionized water/ethanol by sonication, forming a black dispersion. Next, 5 *μ*l of the as-prepared dispersion was casted onto a glass carbon electrode 3 mm in diameter as the working electrode. Meanwhile, a saturated calomel electrode (SCE) was applied as the counter electrode and a Pt sheet was applied as the reference electrode. The typical electrochemically active surface area (ECSA) measurement of our samples was tested in nitrogen-saturated 0.5 M H_2_SO_4_ electrolyte at 10 mV s^−1^. The MOR electrocatalytic activity was recorded in 0.5 M H_2_SO_4_ and 1 M methanol electrolyte at 20 mV s^−1^. For Pd-based catalysts, the MOR electrocatalytic activity was recorded in 1 M KOH and 1 M methanol electrolyte. The chronoamperometry (CA) test was conducted for a period of 1 h at room temperature. The electrochemical impedance spectra (EIS) were recorded at the frequency range from 100000 Hz to 0.01 Hz with an amplitude of 10 mV.

## Supplementary Materials

Supplementary Materials Figure S1: photograph of the synthesis process of 3D GB-Pt scaffolds with (a) 0.3 mg/ml, (b) 0.5 mg/ml, (c) 1 mg/ml, (d) 2 mg/ml, and (e) 3 mg/ml of (NH_4_)_2_PtCl_6_ solution as the Pt precursor. Figure S2: changes of the electronic absorption spectrum of the (NH_4_)_2_PtCl_6_ with a different amount of NaCl, clearly indicating that the [PtCl_6_]^2−^ ion undergoes deep hydrolysis with less amount of NaCl. Figure S3: morphological characterizations of the fast cryoequilibrium hydrolysates. (a–c) TEM and HRTEM images of the fast cryoequilibrium hydrolysates (pH(2)) with an inset image of SAED patterns. (d–f) TEM and HRTEM images of the fast cryoequilibrium hydrolysates (pH(7)) with an inset image of SAED patterns. (g–i) TEM and HRTEM images of the fast cryoequilibrium hydrolysates (pH(12)) with an inset image of SAED patterns. Figure S4: (a, e) STEM images and (b–d and f–h) corresponding elemental mapping images of the fast cryoequilibrium hydrolysates. Figure S5: FESEM images of 3D GB-Pt scaffolds-*X*. (a, b) GB-Pt scaffolds-30. (c, d) GB-Pt scaffolds-20. (e, f) GB-Pt scaffolds-10. (g, h) GB-Pt scaffolds-5. (i, j) GB-Pt scaffolds-3. Figure S6: SEM images of the products obtained by hydrogen reduction of fast cryoequilibrium hydrolysate intermediates with different time: (a) 0 min, (b) 40 min, (c) 80 min, and (d) 120 min. Figure S7: TEM images of 3D GB-Pt scaffolds-*X*. (a, b) GB-Pt scaffolds-30. (c, d) GB-Pt scaffolds-20. (e, f) GB-Pt scaffolds-10. (g, h) GB-Pt scaffolds-5. (i, j) GB-Pt scaffolds-3. Figure S8: (a–d) HRTEM images of 3D GB-Pt scaffolds with marked grain boundaries. Figure S9: morphological characterizations of slow cryogenic hydrolysates. (a–c) TEM and HRTEM images of the slow cryoequilibrium hydrolysates (pH(7)). (d–f) TEM and HRTEM images of the slow cryoequilibrium hydrolysates (pH(2)). Figure S10: (a–c) SEM images of Pt rods. Figure S11: (a–c) TEM images of Pt rods. Figure S12: FESEM images of GB-Pt scaffolds derived from hydrogen reduction of fast cryoequilibrium hydrolysates with different pH values. (a–c) 3D GB-Pt scaffolds-pH(0). (d–f) 3D GB-Pt scaffolds-pH(2). (g–i) 3D GB-Pt scaffolds-pH(7). (j–l) 3D GB-Pt scaffolds-pH(12). (m–o) 3D GB-Pt scaffolds-pH(14). Figure S13: TEM images of GB-Pt scaffolds derived from hydrogen reduction of fast cryoequilibrium hydrolysates with different pH values. (a–c) 3D GB-Pt scaffolds-pH(0). (d–f) 3D GB-Pt scaffolds-pH(2). (g–i) 3D GB-Pt scaffolds-pH(7). (j–l) 3D GB-Pt scaffolds-pH(12). (m–o) 3D GB-Pt scaffolds-pH(14). Figure S14: SEM images of reduced fast cryoequilibrium hydrolysates of K_2_PtCl_6_ without and with additional NH_4_Cl. (a-c) SEM images of reduced fast cryoequilibrium hydrolysates of K_2_PtCl_6_ solution. (d–f) SEM images of reduced fast cryoequilibrium hydrolysates of K_2_PtCl_6_ and NH_4_Cl solution. Figure S15: (a–c) TEM images of reduced fast cryoequilibrium hydrolysates of K_2_PtCl_6_ with additional NH4Cl, with marked grain boundaries in (c). Figure S16: SEM images of reduced fast cryoequilibrium hydrolysates of Na_2_PtCl_6_ without and with additional NH_4_Cl. (a–c) SEM images of reduced fast cryoequilibrium hydrolysates of Na_2_PtCl_6_ solution. (d–f) SEM images of reduced fast cryoequilibrium hydrolysates of Na_2_PtCl_6_ and NH_4_Cl solution. Figure S17: (a–c) TEM images of reduced fast cryoequilibrium hydrolysates of Na_2_PtCl_6_ with additional NH_4_Cl, with marked grain boundaries in (c). Figure S18: FESEM and TEM images of 3D GB-Pd scaffolds. (a, b) FESEM image of 3D GB-Pd scaffolds with different magnifications. (c, d) TEM images of 3D GB-Pd scaffolds with different magnifications. (e) HRTEM images of 3D GB-Pd scaffolds, exhibiting *d*-spacing values of 0.234 nm (Pd (111) lattices). (f) SAED patterns of 3D GB-Pd scaffolds. Figure S19: (a–d) HRTEM images of 3D GB-Pd scaffolds with marked grain boundaries. Figure S20: XRD patterns of the 3D GB-Pd scaffolds and Pd/C. Figure S21: (a) nitrogen adsorption/desorption isotherm of 3D GB-Pd scaffolds. (b) Nitrogen adsorption/desorption isotherm of Pd/C. Figure S22: grain length distribution of the 3D GB-Pt scaffolds. (a–e) Grain length distribution of GB-Pt scaffolds-3, GB-Pt scaffolds-5, GB-Pt scaffolds-10, GB-Pt scaffolds-20, and GB-Pt scaffolds-30, respectively. (f) Average grain length and grain boundary density of the 3D GB-Pt scaffolds. Figure S23: grain length distribution of the 3D GB-Pd scaffolds. Figure S24: XRD patterns of the 3D GB-Pt scaffolds. Figure S25: XRD patterns of the 3D GB-Pt scaffolds prepared from different pH values of the (NH_4_)_2_PtCl_6_ solution. Figure S26: XPS spectra of the 3D GB-Pt scaffolds. Figure S27: Pt 4f spectra of the 3D GB-Pt scaffolds, Pt rods, and Pt/C samples. Figure S28: cyclic voltammetry curves of the 3D GB-Pt scaffolds in the 0.5 M H_2_SO_4_ electrolyte at a sweep rate of 10 mV s^−1^. Figure S29: electrocatalytic MOR properties of (a) 3D GB-Pt scaffolds and (b) Pt rods and Pt/C catalysts. Figure S30: electrocatalytic MOR properties of the 3D GB-Pt scaffolds-pH(0), (2), (7), (12), and (14). Figure S31: CO tolerance of 3D GB-Pt scaffold and Pt/C catalyst measurement by stripping voltammetry of adsorbed CO, indicating that 3D GB-Pt scaffolds have enhanced antipoisoning (CO) property. Figure S32: (a) TEM and (b) HRTEM images of 3D GB-Pt scaffolds after 5000 cycles. Figure S33: Nyquist plots for the 3D GB-Pt scaffolds, Pt rods, and Pt/C electrocatalysts. (a–c) Nyquist plots for (a) 3D GB-Pt scaffolds, (b) Pt rods, and (c) Pt/C in electrochemical methanol oxidation at different potentials. (d) Nyquist plots of 3D GB-Pt scaffolds, Pt rods, and Pt/C electrocatalysts for methanol oxidation at 0.4 V. Figure S34: (a–c) Nyquist plots of 3D GB-Pt scaffolds, Pt rods, and Pt/C electrocatalysts for methanol oxidation before and after stability test. Figure S35: (a, b) comparative Pd specific activity and mass activity of 3D GB-Pd scaffolds and Pd/C (10 wt%) in 1 M KOH and 1 M methanol electrolyte. Figure S36: CVs of 3D GB-Pd scaffolds before and after 5000 cycles in 1 M KOH and 1 M methanol electrolyte. Table S1: electrochemical surface area (ECSA) of 3D GB-Pt scaffolds, Pt rods, and commercial Pt/C electrocatalysts.
