# Supplementary material for: Fast Cryomediated Dynamic Equilibrium Hydrolysates towards Grain Boundary-Enriched Platinum Scaffolds for Efficient Methanol Oxidation
Source: Research (Wash D C). 2019 Oct 13;2019:8174314. doi: 10.34133/2019/8174314 (PMC6946255; doi:10.34133/2019/8174314)
Supplement: Supplementary Materials — Figure S1: photograph of the synthesis process of 3D GB-Pt scaffolds with (a) 0.3 mg/ml, (b) 0.5 mg/ml, (c) 1 mg/ml, (d) 2 mg/ml, and (e) 3 mg/ml of (NH4)2PtCl6 solution as the Pt precursor. Figure S2: changes of the electronic absorption spectrum of the (NH4)2PtCl6 with a different amount of NaCl, clearly indicating that the [PtCl6]2− ion undergoes deep hydrolysis with less amount of NaCl. Figure S3: morphological characterizations of the fast cryoequilibrium hydrolysates. (a–c) TEM and HRTEM images of the fast cryoequilibrium hydrolysates (pH(2)) with an inset image of SAED patterns. (d–f) TEM and HRTEM images of the fast cryoequilibrium hydrolysates (pH(7)) with an inset image of SAED patterns. (g–i) TEM and HRTEM images of the fast cryoequilibrium hydrolysates (pH(12)) with an inset image of SAED patterns. Figure S4: (a, e) STEM images and (b–d and f–h) corresponding elemental mapping images of the fast cryoequilibrium hydrolysates. Figure S5: FESEM images of 3D GB-Pt scaffolds-X. (a, b) GB-Pt scaffolds-30. (c, d) GB-Pt scaffolds-20. (e, f) GB-Pt scaffolds-10. (g, h) GB-Pt scaffolds-5. (i, j) GB-Pt scaffolds-3. Figure S6: SEM images of the products obtained by hydrogen reduction of fast cryoequilibrium hydrolysate intermediates with different time: (a) 0 min, (b) 40 min, (c) 80 min, and (d) 120 min. Figure S7: TEM images of 3D GB-Pt scaffolds-X. (a, b) GB-Pt scaffolds-30. (c, d) GB-Pt scaffolds-20. (e, f) GB-Pt scaffolds-10. (g, h) GB-Pt scaffolds-5. (i, j) GB-Pt scaffolds-3. Figure S8: (a–d) HRTEM images of 3D GB-Pt scaffolds with marked grain boundaries. Figure S9: morphological characterizations of slow cryogenic hydrolysates. (a–c) TEM and HRTEM images of the slow cryoequilibrium hydrolysates (pH(7)). (d–f) TEM and HRTEM images of the slow cryoequilibrium hydrolysates (pH(2)). Figure S10: (a–c) SEM images of Pt rods. Figure S11: (a–c) TEM images of Pt rods. Figure S12: FESEM images of GB-Pt scaffolds derived from hydrogen reduction of fast cryoequilibrium hy [file 8174314.f1.docx]

Supplementary Materials

Fast Cryo-Mediated Dynamic Equilibrium Hydrolysates towards Grain Boundaries Enriched Platinum Scaffolds for Efficient Methanol Oxidation

Chao Zhang^1^, Huajie Huang^2^, Jianan Gu^1^, Zhiguo Du^1^, Bin Li^1^, Songmei Li^1,^* and Shubin Yang^1,^*

^1^Key Laboratory of Aerospace Advanced Materials and Performance of Ministry of Education, School of Materials Science and Engineering, Beihang University, 100191, Beijing, China

^2^College of Mechanics and Materials, Hohai University, Nanjing 210098, China

*Correspondence to: [songmei_li@buaa.edu.cn](mailto:songmei_li@buaa.edu.cn); [yangshubin@buaa.edu.cn](mailto:yangshubin@buaa.edu.cn)

**Grain boundaries density calculation**

The grain boundaries density of 3D GB-Pt scaffolds was calculated as the following:

$$\mathbf{g}\mathbf{rain boundaries density=}\frac{\mathbf{1}}{\boldsymbol{l}_{\boldsymbol{average}}}$$

Among them, *l*_average_ was the average grain size of single-crystal Pt, which confirmed by counting the size of randomly 200 nanocrystals, with the aid of HRTEM measurement. The grain boundaries density of the samples was defined as unit length of the sample (1 μm) divided by the *l*_average_ of the single-crystal Pt, thus the unit of the density was 1/μm.


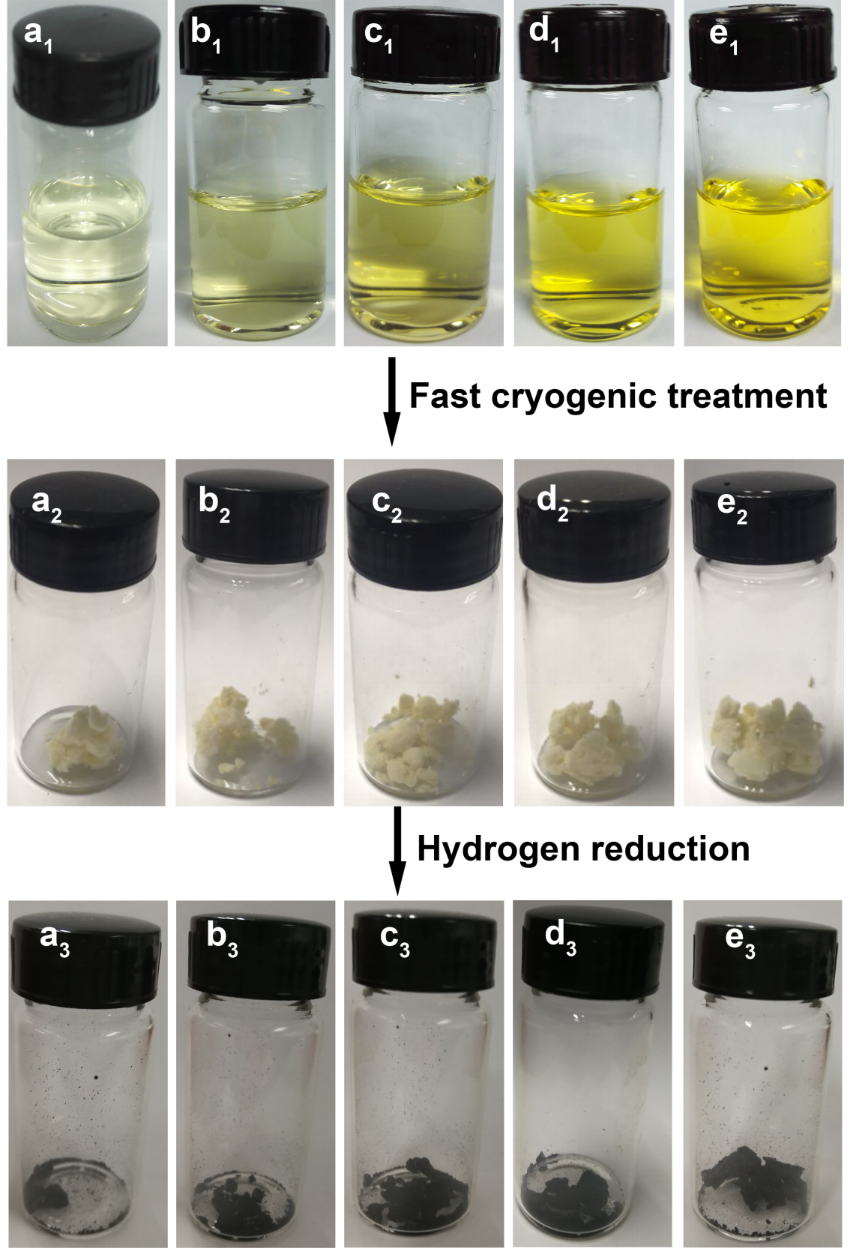


**Figure S1.** Photograph of the synthesis process of 3D GB-Pt scaffolds with (a) 0.3 mg/ml, (b) 0.5 mg/ml, (c) 1 mg/ml, (d) 2 mg/ml and (e) 3 mg/ml of (NH_4_)_2_PtCl_6_ solution as the Pt precursor.


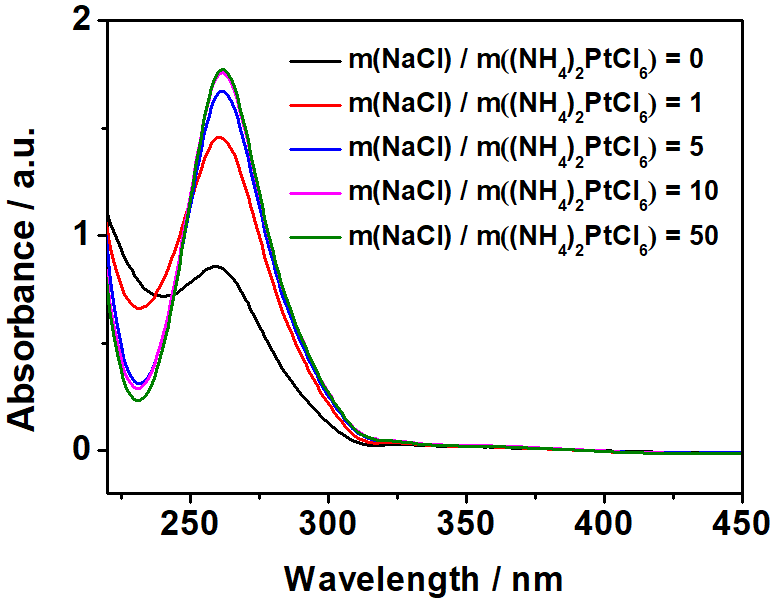


**Figure S2.** Changes of the electronic absorption spectrum of the (NH_4_)_2_PtCl_6_ with different amount of NaCl, clearly indicating that [PtCl_6_]^2-^ ion undergoes deep hydrolysis with less amount of NaCl.


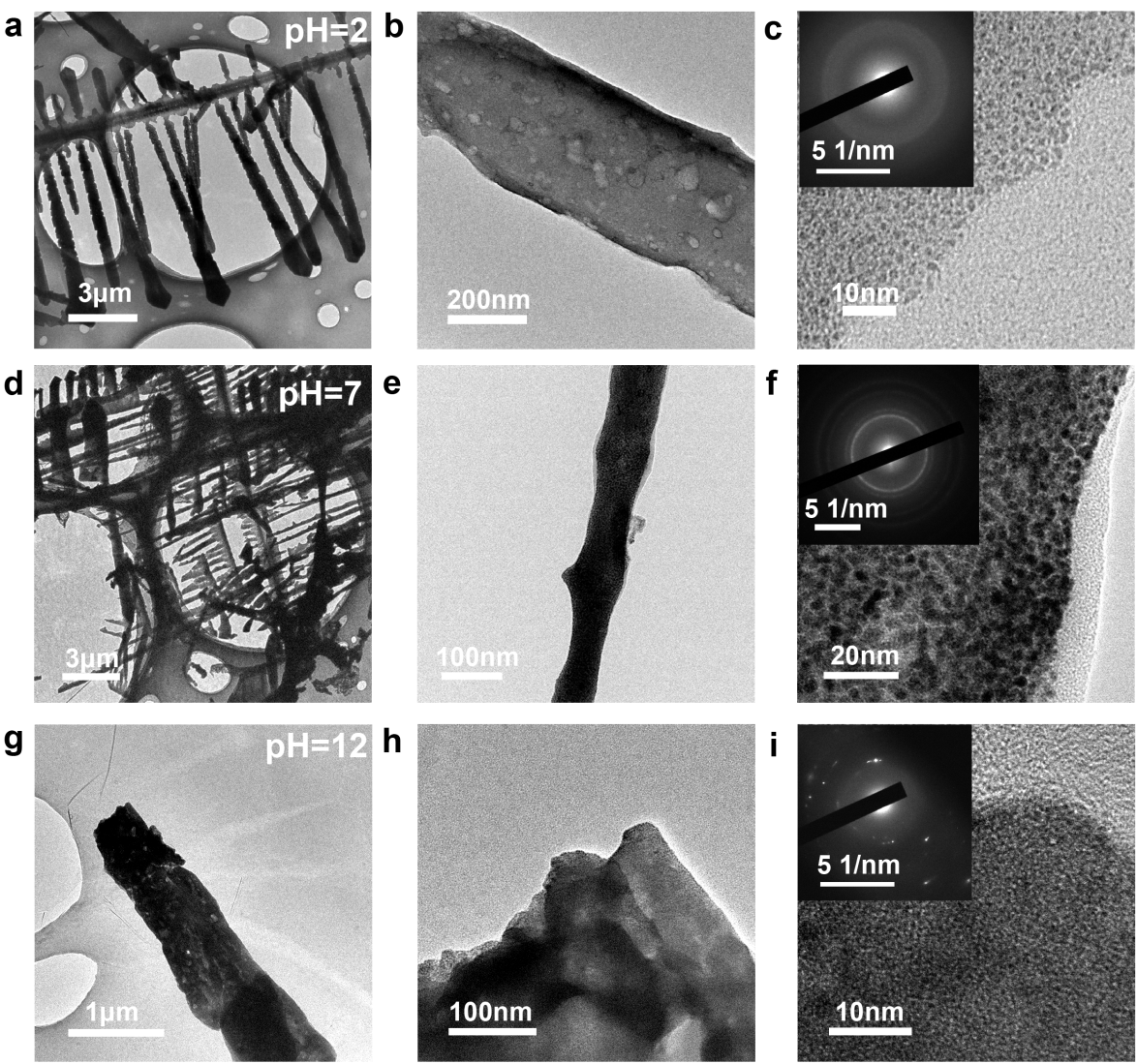


**Figure S3.** Morphological characterizations of the fast cryo-equilibrium-hydrolysates. (a-c) TEM and HRTEM images of the fast cryo-equilibrium-hydrolysates (pH(2)) with inset image of SAED patterns. (d-f) TEM and HRTEM images of the fast cryo-equilibrium-hydrolysates (pH(7)) with inset image of SAED patterns. (g-i) TEM and HRTEM images of the fast cryo-equilibrium-hydrolysates (pH(12)) with inset image of SAED patterns. As shown in Figure S3d and e, after fast cryogenic treatment of neutral (NH_4_)_2_PtCl_6_ (pH(7)) solution, the intermediates have intertwined rod shape with size of nearly 20 μm in length and 0.1-2 μm in diameter. HRTEM images (Figure S3f) show that many Pt-containing intermediates nanodots with diameters of 3-5 nm are homogeneously encapsulated in the microrod. Interestingly, the microrod are encapsulated by a thin shell with a thickness of nearly 20 nm, corresponding to NH_4_Cl. In comparison, in the case of fast cryo-equilibrium-hydrolysates at a pH value of 2, the shell is much thinner than that at a pH value of 7. This should be ascribed to the thinner NH_4_Cl layer (Figure S3a-c), whose formation was inhibited in the acidic condition. On the contrary, under alkaline condition (pH=12), the fast cryo-equilibrium-hydrolysates are big particles (Figure S3g-i). This should be originated to the presence of excess NH_4_Cl in the alkaline condition that prevent the connection of the intermediates. Select area electron diffraction (SAED) patterns in Figure S3f reveal the amorphous structure of the fast cryo-equilibrium-hydrolysates intermediates.


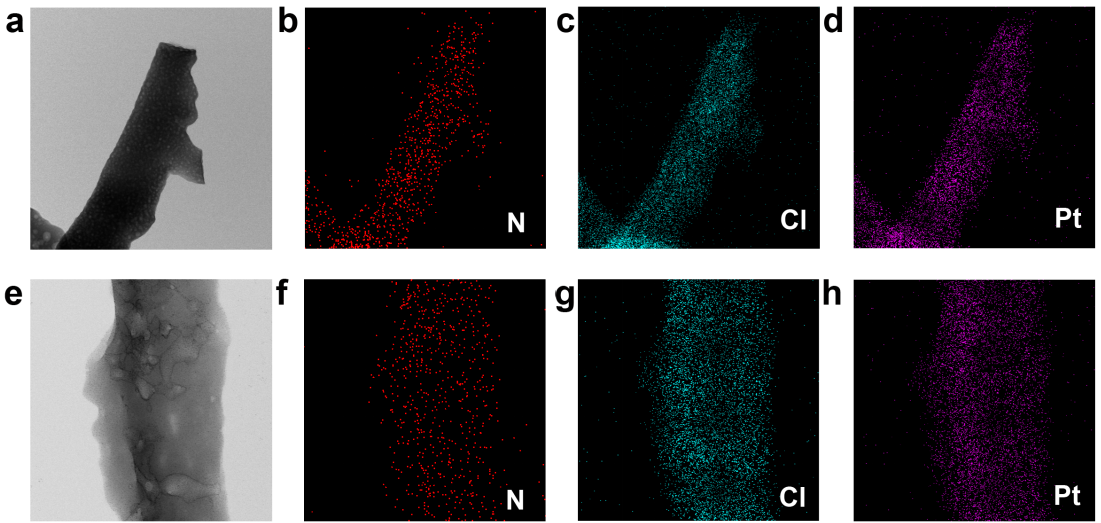


**Figure S4.** (a and e) STEM images and (b-d and f-h) corresponding elemental mapping images of the fast cryo-equilibrium-hydrolysates.


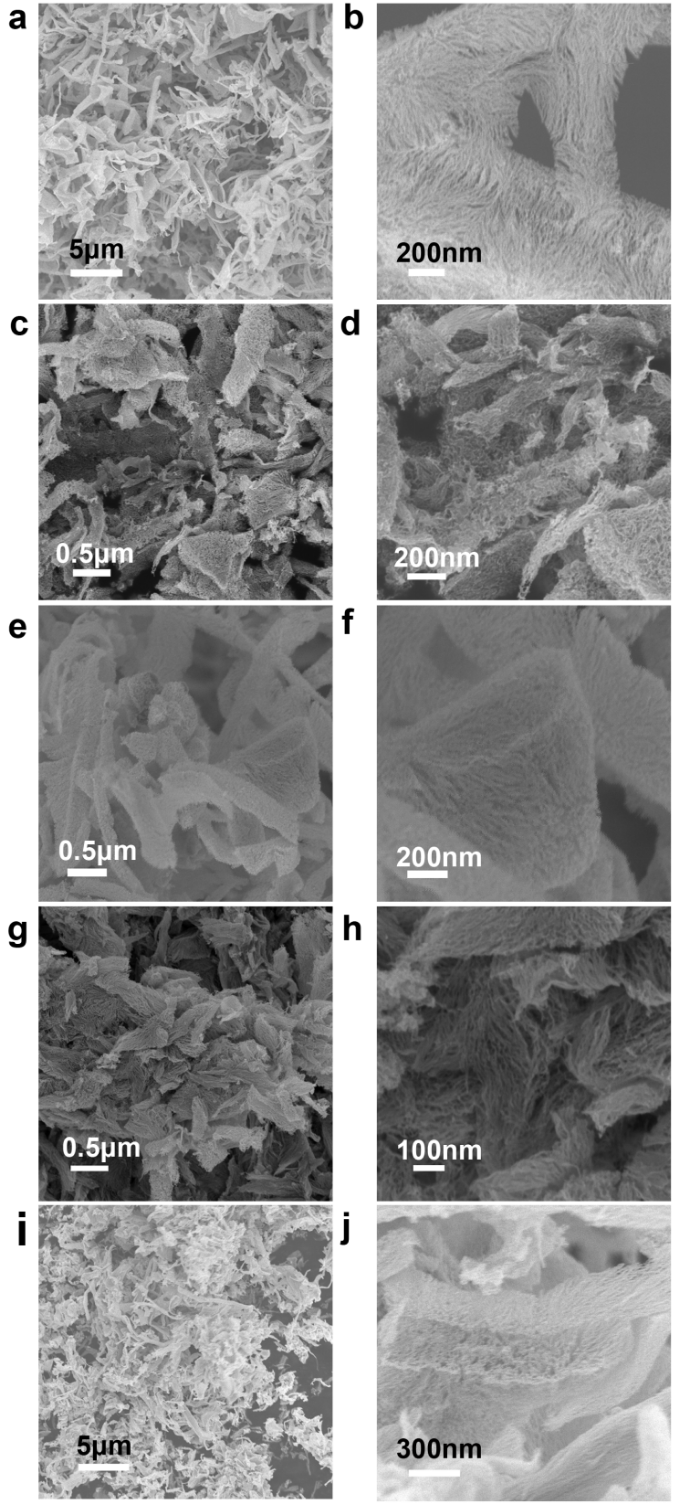


**Figure S5.** FESEM images of 3D GB-Pt scaffolds-X. (a and b) GB-Pt scaffolds-30. (c and d) GB-Pt scaffolds-20. (e and f) GB-Pt scaffolds-10. (g and h) GB-Pt scaffolds-5. (i and j) GB-Pt scaffolds-3.


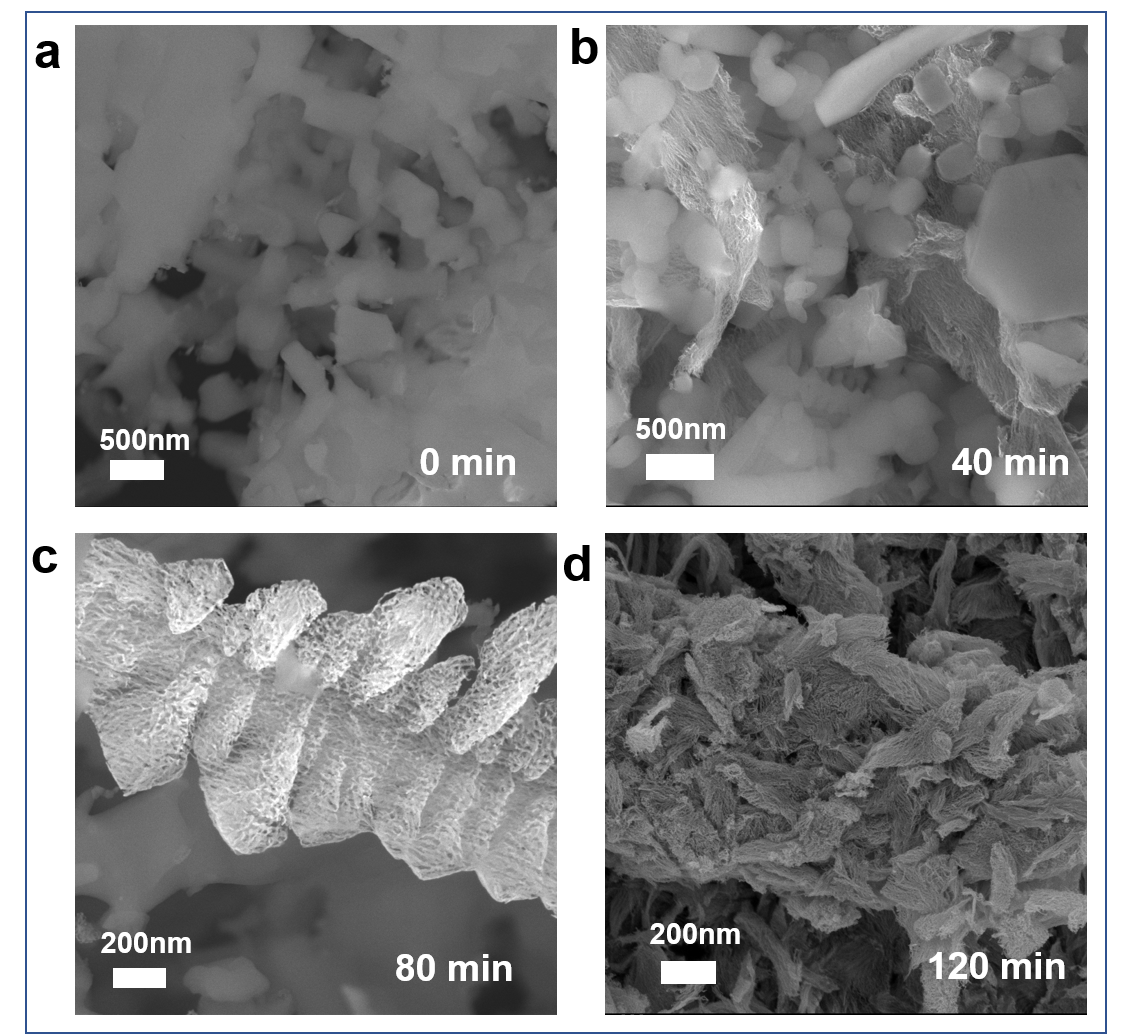


**Figure S6.** SEM images of the products obtained by hydrogen reduction of fast cryo-equilibrium-hydrolysates intermediates with different time: (a) 0 min, (b) 40 min, (c) 80 min, (d) 120 min. During the reduction process, numerous individual Pt nanocrystallines could be derived from Pt-containing intermediates, and at the same time NH_4_Cl would be gradually decomposed and left gaps between the Pt nanocrystallines. Such gaps allow to the infusion between adjacent Pt nanocrystallines, generating grain boundaries enriched GB-Pt scaffolds.


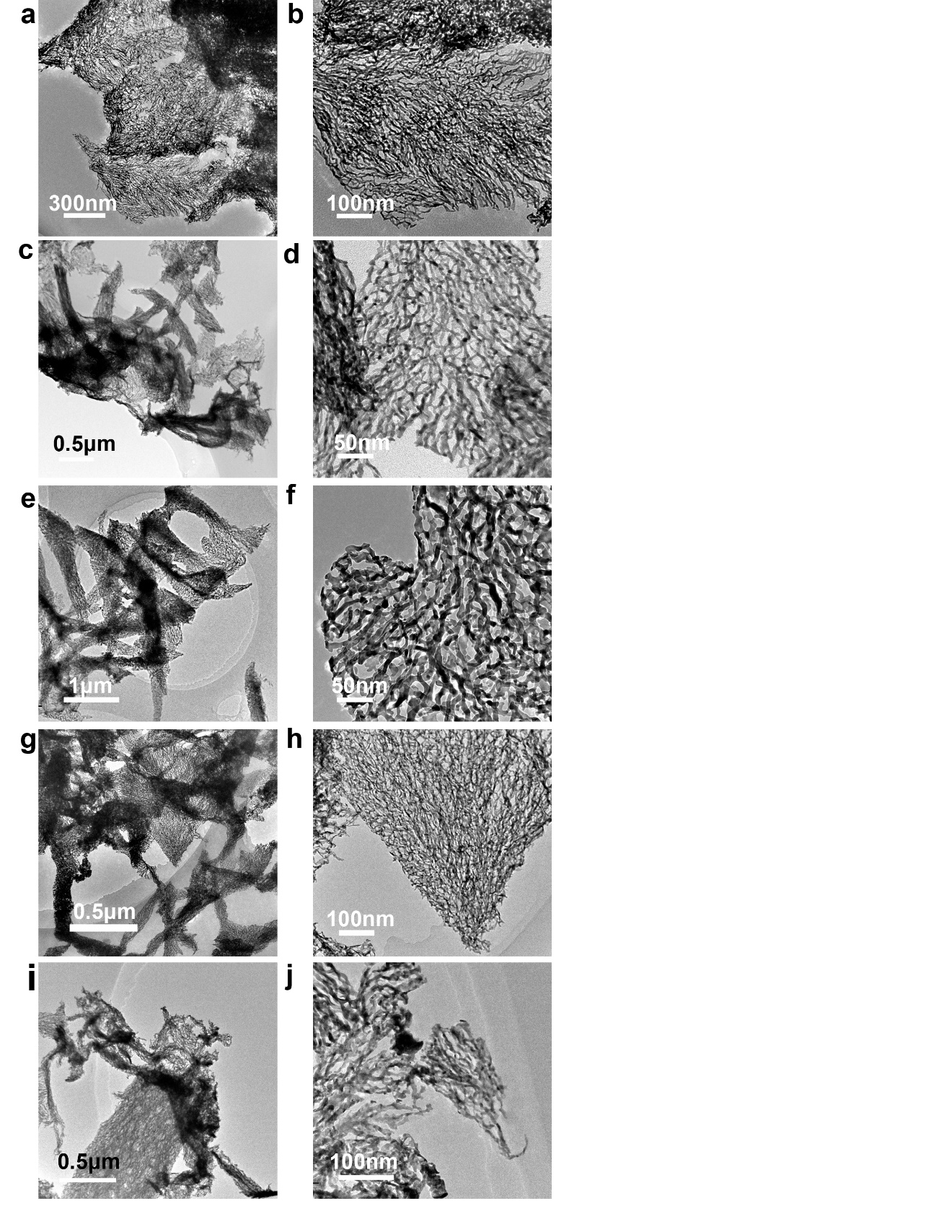


**Figure S7.** TEM images of 3D GB-Pt scaffolds-X. (a and b) GB-Pt scaffolds-30. (c and d) GB-Pt scaffolds-20. (e and f) GB-Pt scaffolds-10. (g and h) GB-Pt scaffolds-5. (i and j) GB-Pt scaffolds-3.


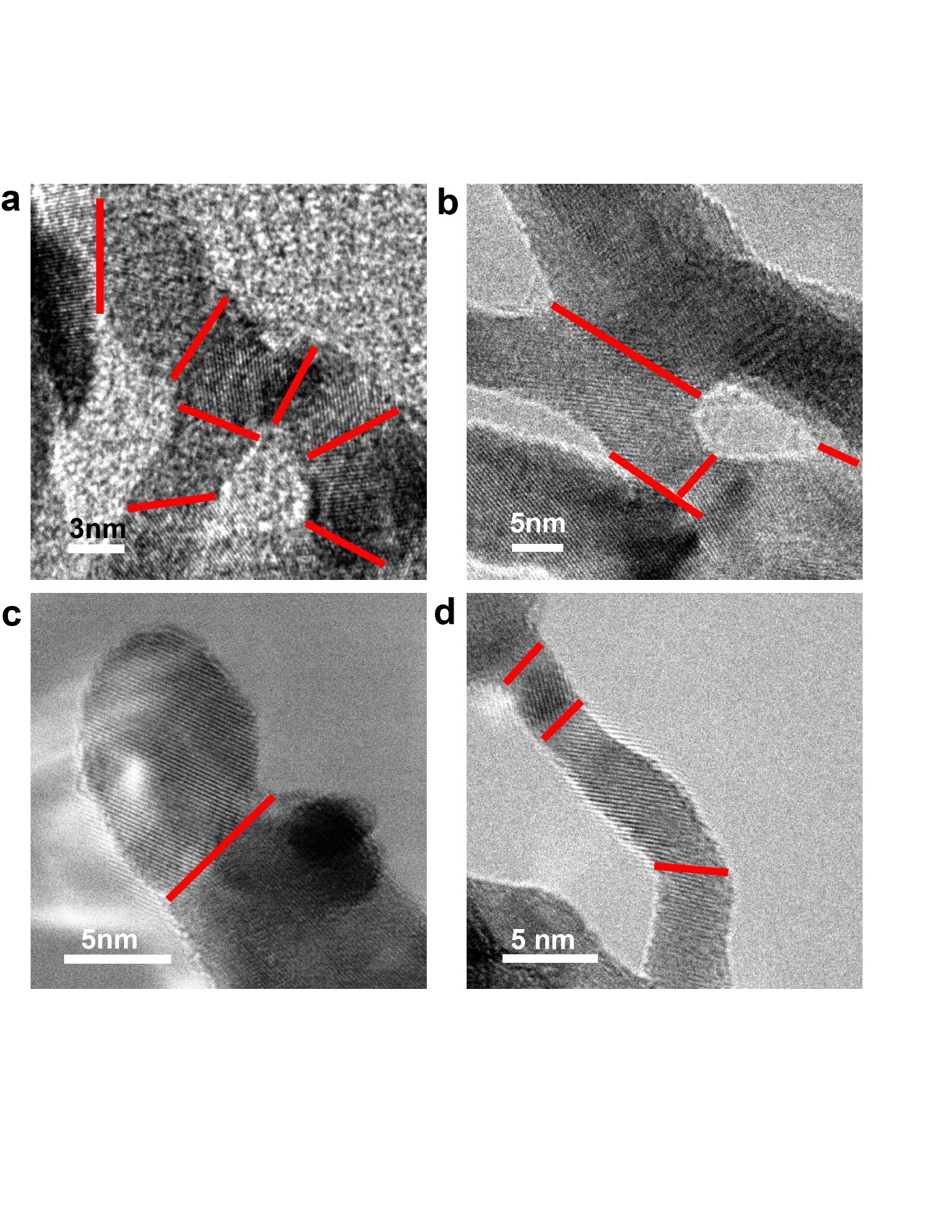


**Figure S8.** (a-d) HRTEM images of 3D GB-Pt scaffolds with marked grain boundaries.


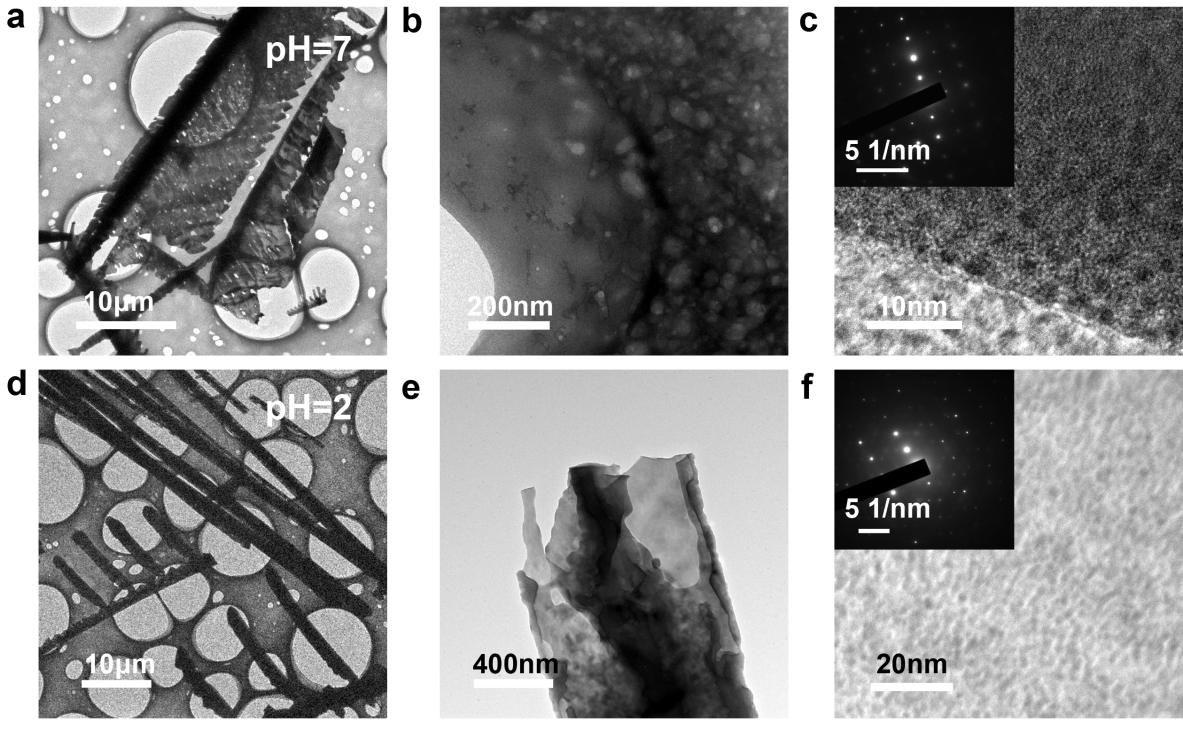


**Figure S9.** Morphological characterizations of slow cryogenic hydrolysates. (a-c) TEM and HRTEM images of the slow cryo-equilibrium-hydrolysates (pH(7)). (d-f) TEM and HRTEM images of the slow cryo-equilibrium-hydrolysates (pH(2)). Clearly, slow cryogenic treatment results in re-crystallization of the (NH_4_)_2_PtCl_6_. It is confirmed that the different levels of hydrolysate [PtCl_6-n_(OH)_n_]^2-^ were re-crystallized to single-crystalline (NH_4_)_2_PtCl_6_ during the slow cryogenic treatment process (SAED results inset in Figure S9c).


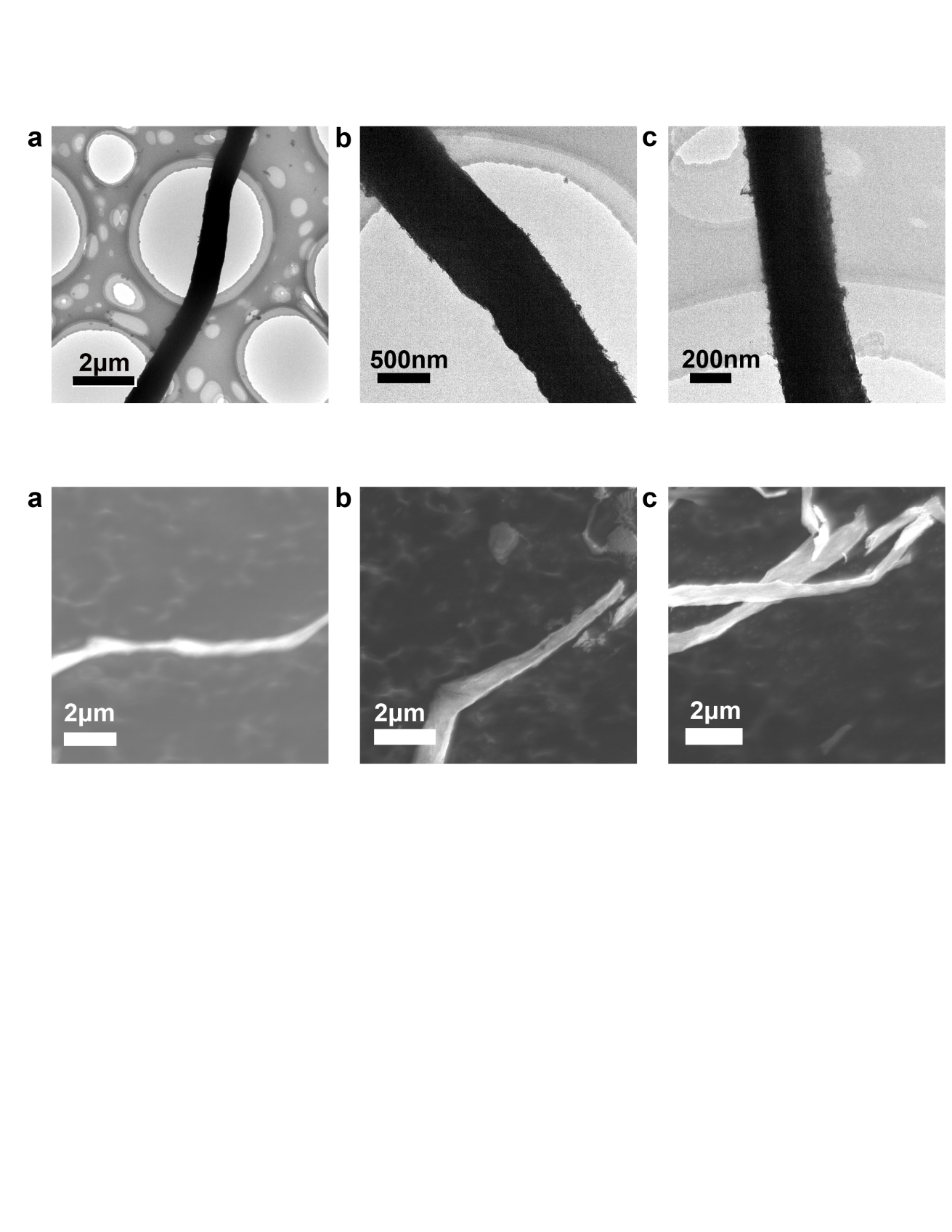


**Figure S10.** (a-c) SEM images of Pt rods.


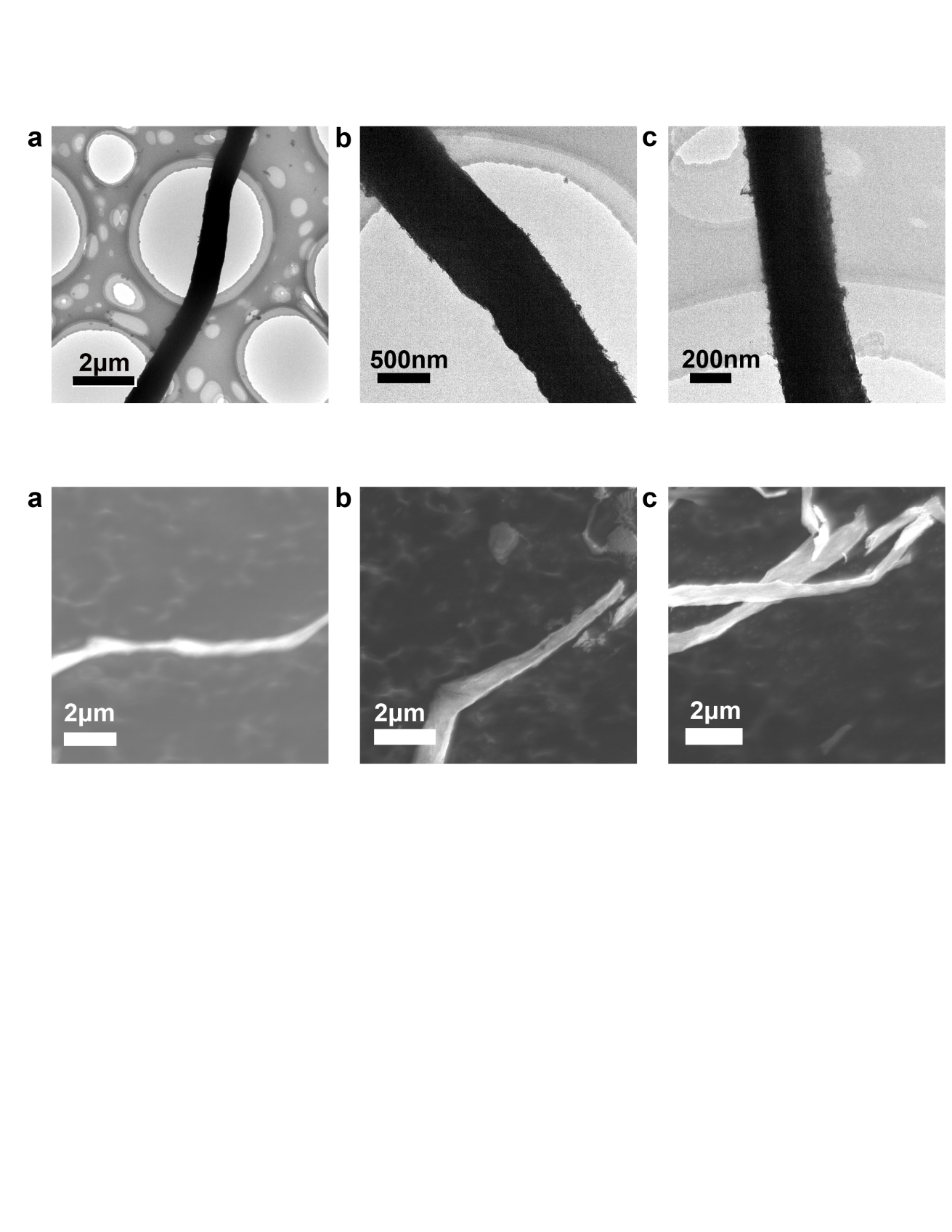


**Figure S11.** (a-c) TEM images of Pt rods.


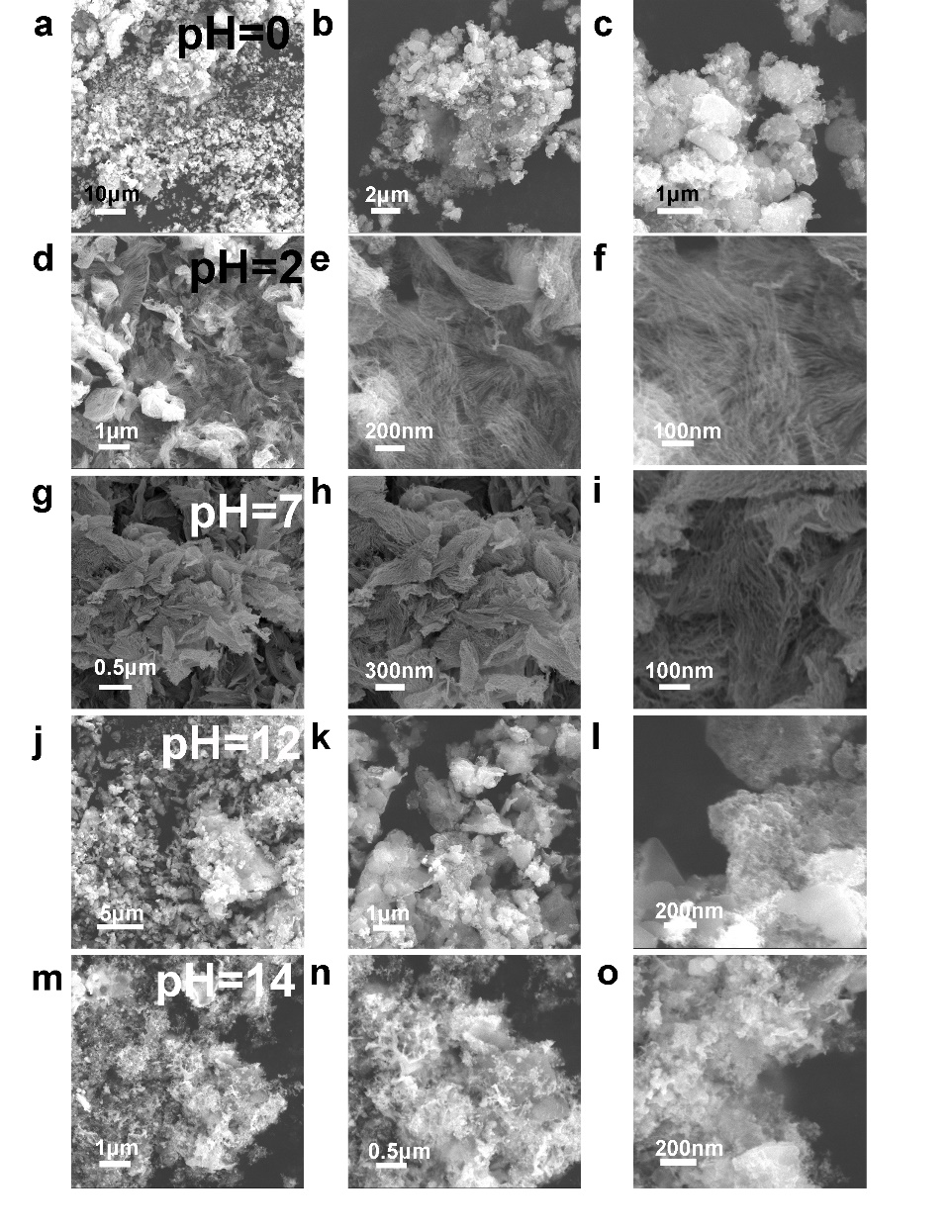


**Figure S12.** FESEM images of GB-Pt scaffolds derived from hydrogen reduction of fast cryo-equilibrium-hydrolysates with different pH values. (a-c) 3D GB-Pt scaffolds-pH(0). (d-f) 3D GB-Pt scaffolds-pH(2). (g-i) 3D GB-Pt scaffolds-pH(7). (j-l) 3D GB-Pt scaffolds-pH(12). (m-o) 3D GB-Pt scaffolds-pH(14).


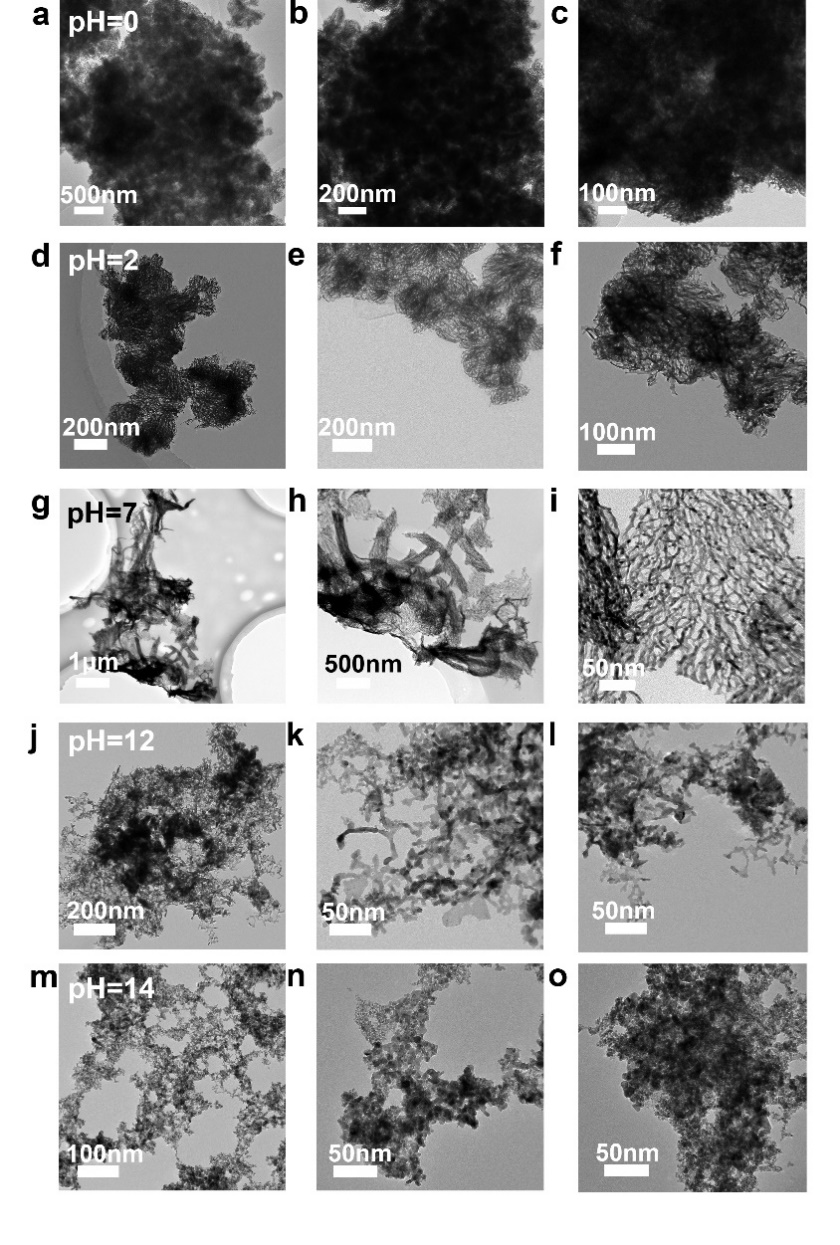


**Figure S13.** TEM images of GB-Pt scaffolds derived from hydrogen reduction of fast cryo-equilibrium-hydrolysates with different pH values. (a-c) 3D GB-Pt scaffolds-pH(0). (d-f) 3D GB-Pt scaffolds-pH(2). (g-i) 3D GB-Pt scaffolds-pH(7). (j-l) 3D GB-Pt scaffolds-pH(12). (m-o) 3D GB-Pt scaffolds-pH(14). As shown in Figure S12 and S13, while the pH value is 2 and 7, the resultant GB-Pt scaffolds show interconnected 3D networks. As pH value is 0, all the nanowires or particles are strongly aggregated, owing to the less amount of hydrolysis product NH_4_Cl, which can’t efficiently prevent the aggregation during reduction process. In contrast, as pH value arrives to 12 and 14, all the Pt nanocrystallines are separated, without formation of good networks.


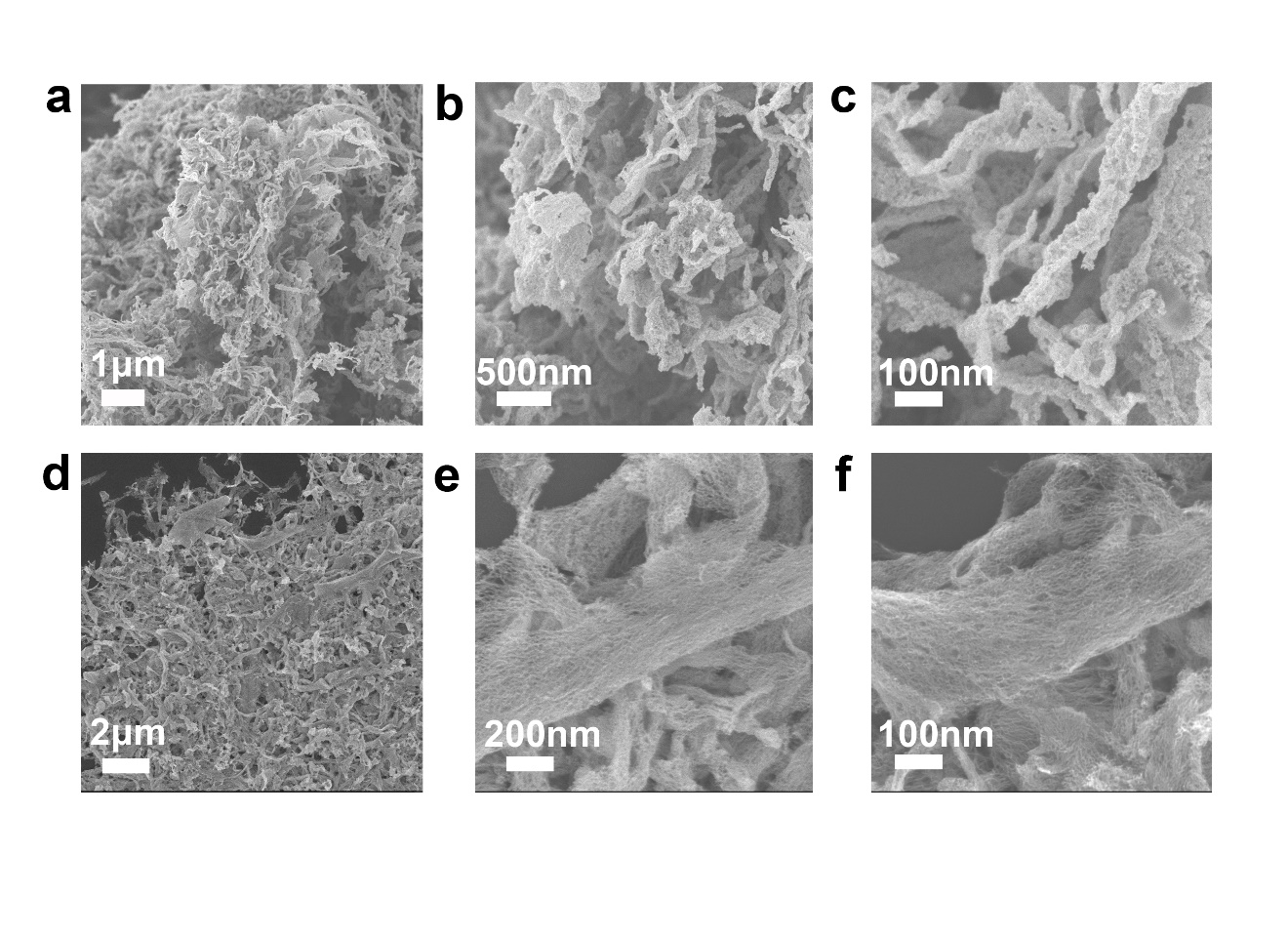


**Figure S14.** SEM images of reduced fast cryo-equilibrium-hydrolysates of K_2_PtCl_6_ without and with additional NH_4_Cl. (a-c) SEM images of reduced fast cryo-equilibrium-hydrolysates of K_2_PtCl_6_ solution. (d-f) SEM images of reduced fast cryo-equilibrium-hydrolysates of K_2_PtCl_6_ and NH_4_Cl solution.


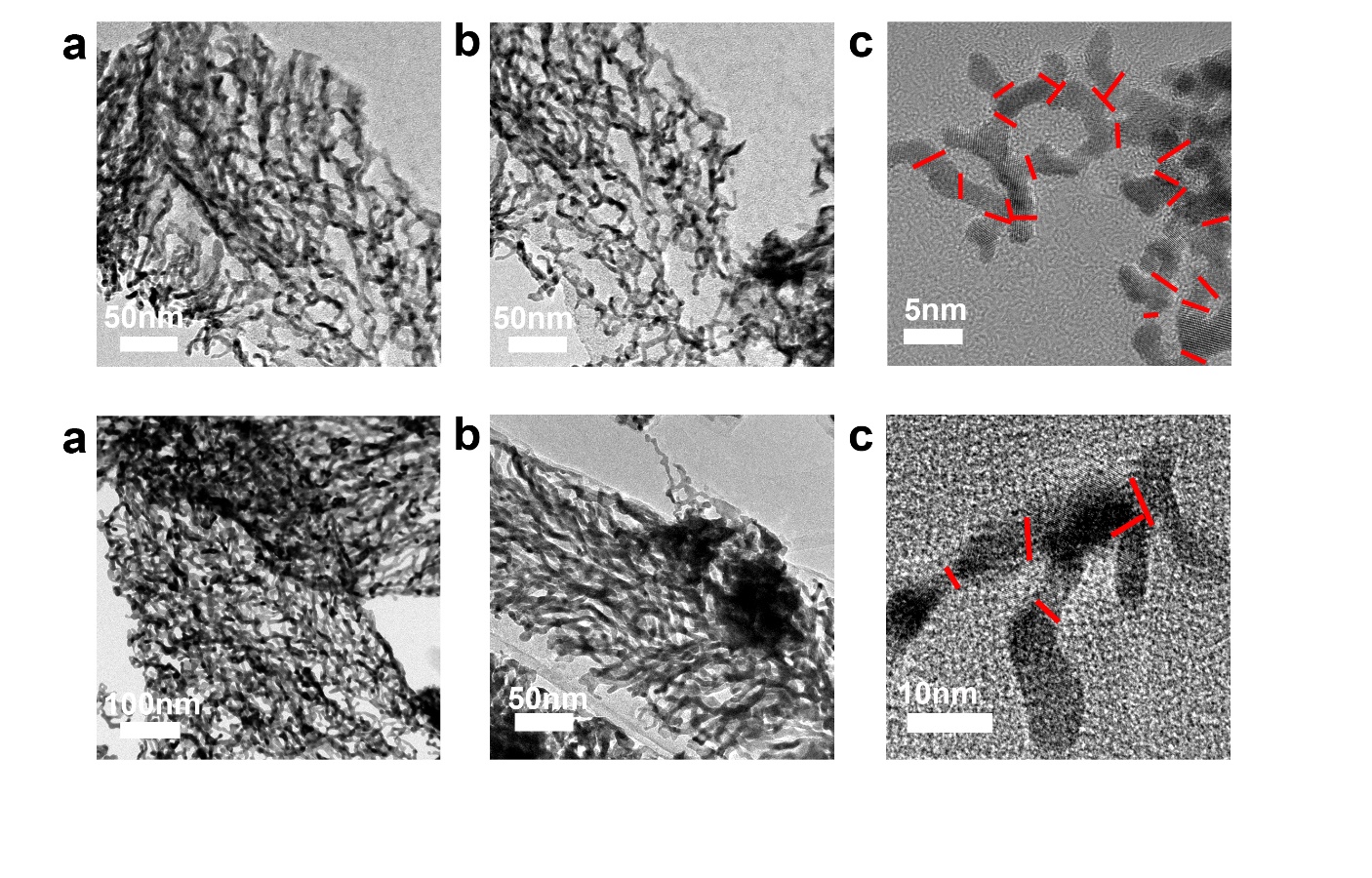


**Figure S15.** (a-c) TEM images of reduced fast cryo-equilibrium-hydrolysates of K_2_PtCl_6_ with additional NH_4_Cl, with marked grain boundaries in (c).


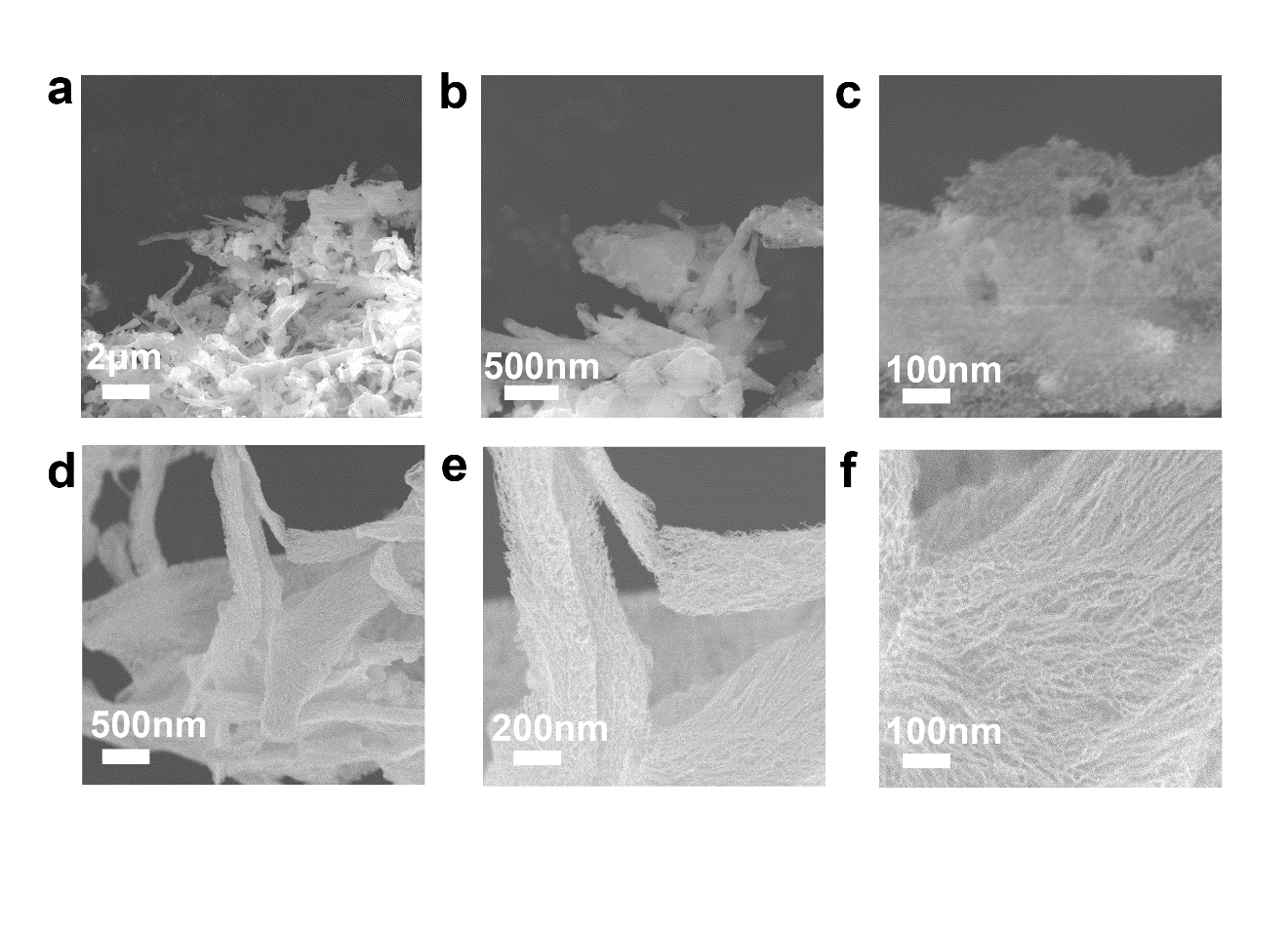


**Figure S16.** SEM images of reduced fast cryo-equilibrium-hydrolysates of Na_2_PtCl_6_ without and with additional NH_4_Cl. (a-c) SEM images of reduced fast cryo-equilibrium-hydrolysates of Na_2_PtCl_6_ solution. (d-f) SEM images of reduced fast cryo-equilibrium-hydrolysates of Na_2_PtCl_6_ and NH_4_Cl solution.


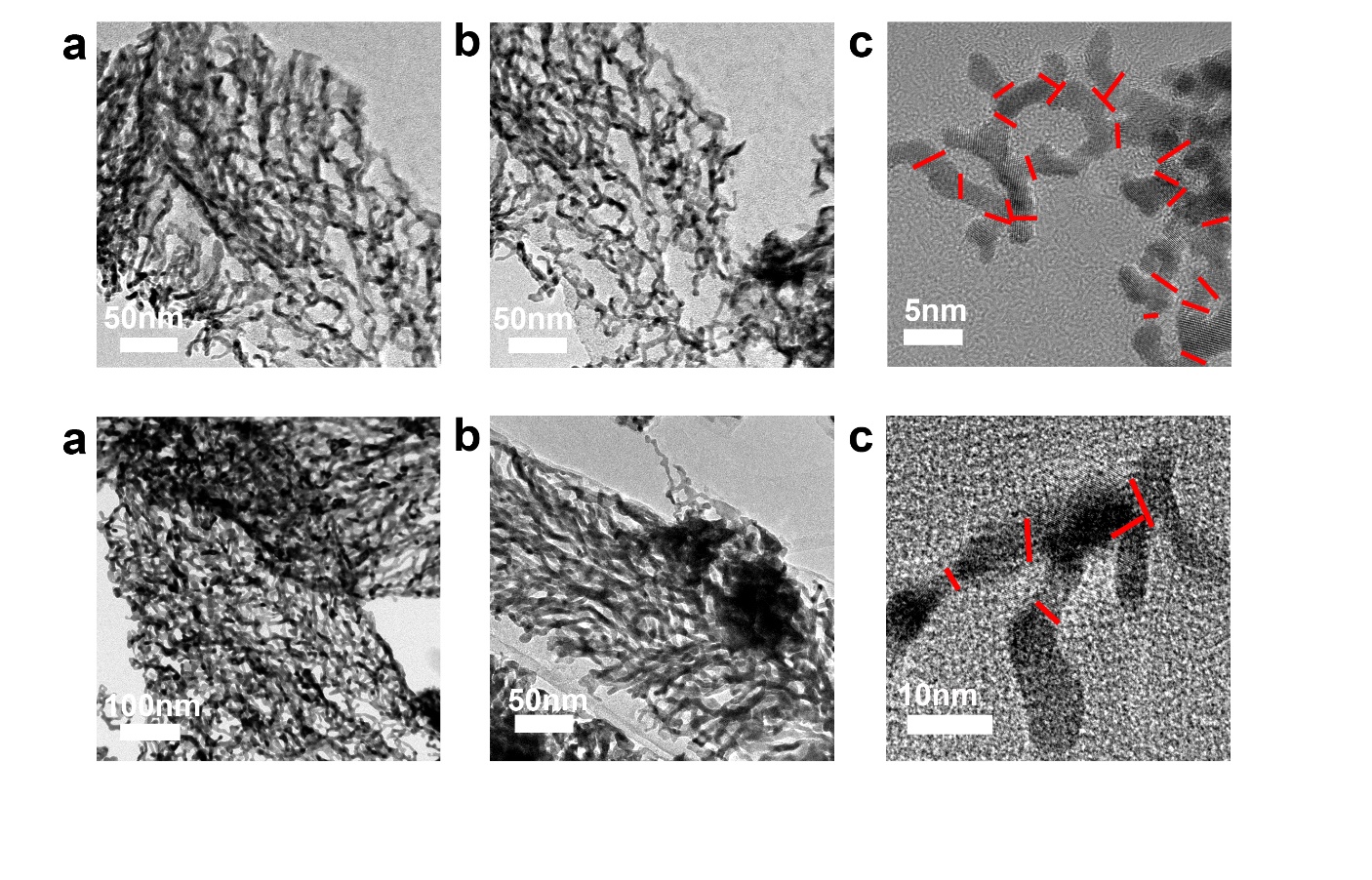


**Figure S17.** (a-c) TEM images of reduced fast cryo-equilibrium-hydrolysates of Na_2_PtCl_6_ with additional NH_4_Cl, with marked grain boundaries in (c).


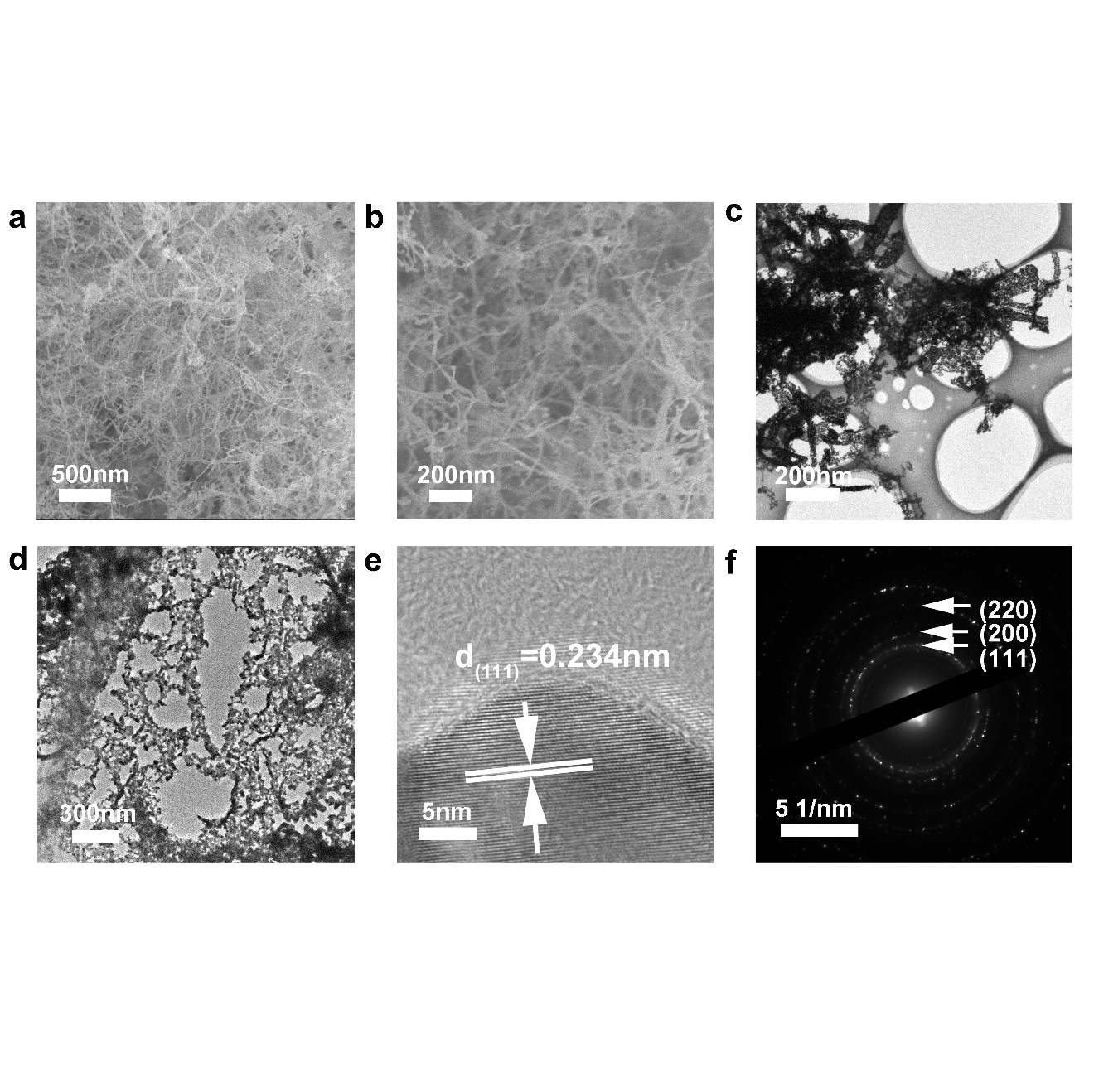


**Figure S18.** FESEM and TEM images of 3D GB-Pd scaffolds. (a and b) FESEM image of 3D GB-Pd scaffolds with different magnifications. (c and d) TEM images of 3D GB-Pd scaffolds with different magnifications. (e) HRTEM images of 3D GB-Pd scaffolds, exhibiting a d-spacing values of 0.234 nm (Pd (111) lattices). (f) SAED patterns of 3D GB-Pd scaffolds.


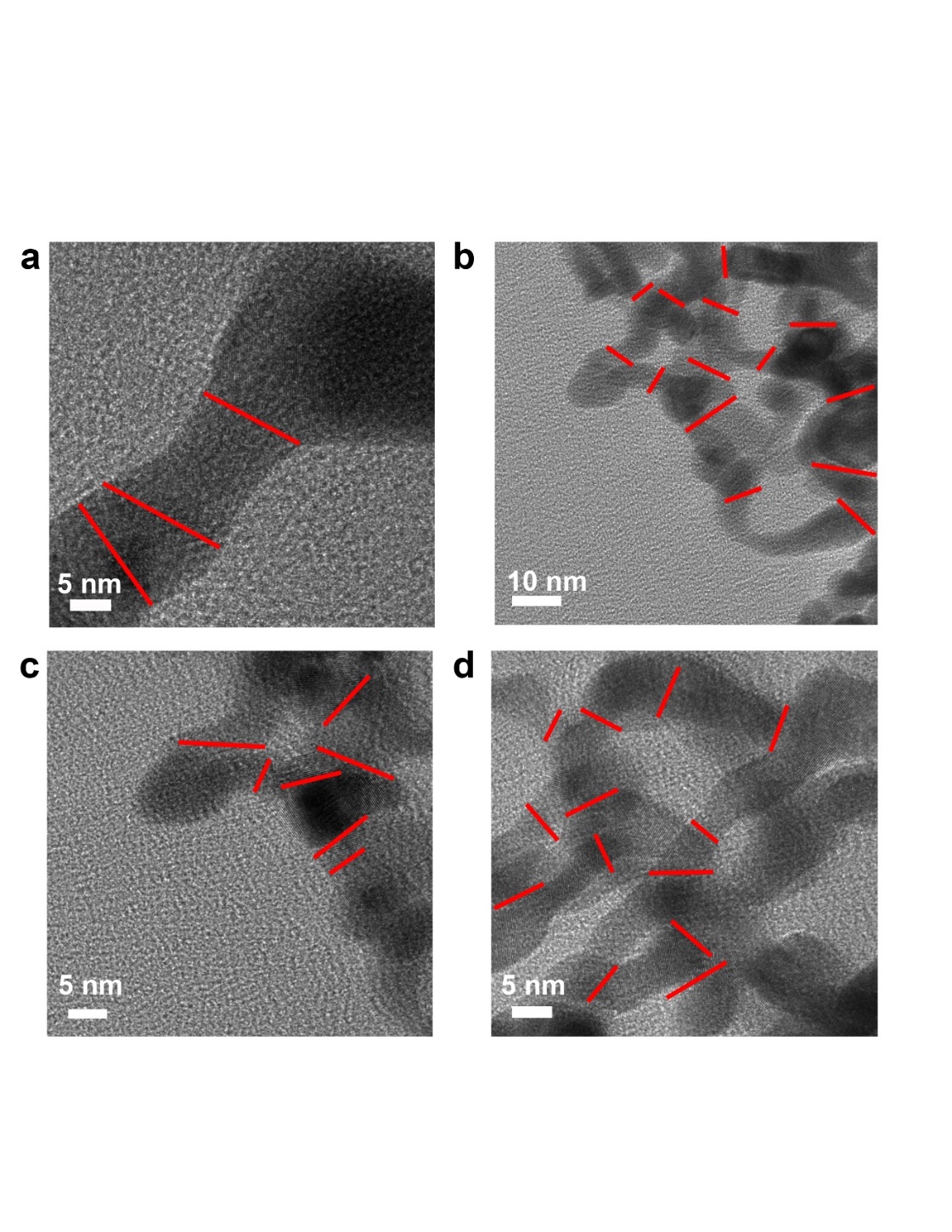


**Figure S19.** (a-d) HRTEM images of 3D GB-Pd scaffolds with marked grain boundaries.


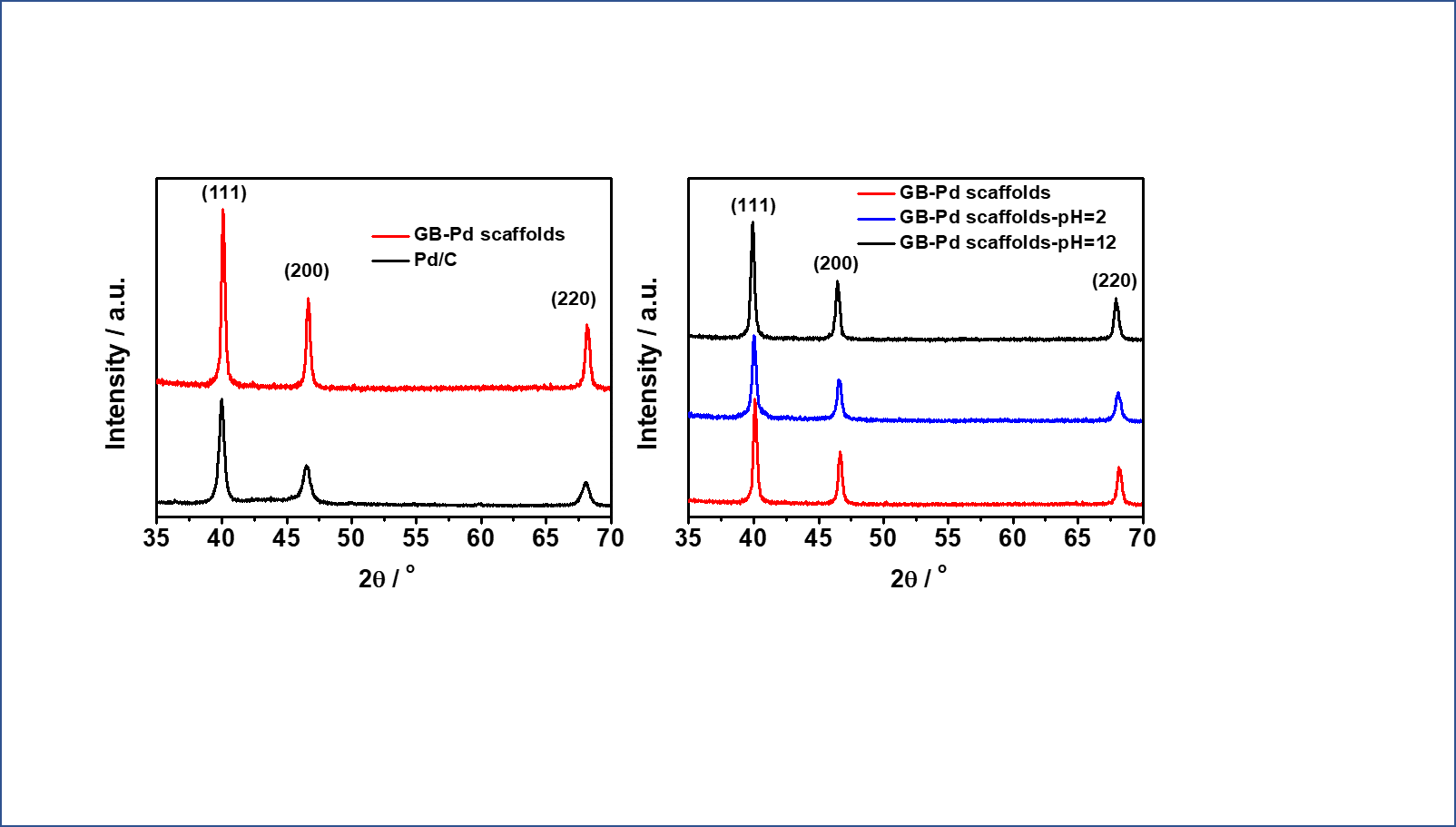


**Figure S20.** XRD patterns of the 3D GB-Pd scaffolds and Pd/C.


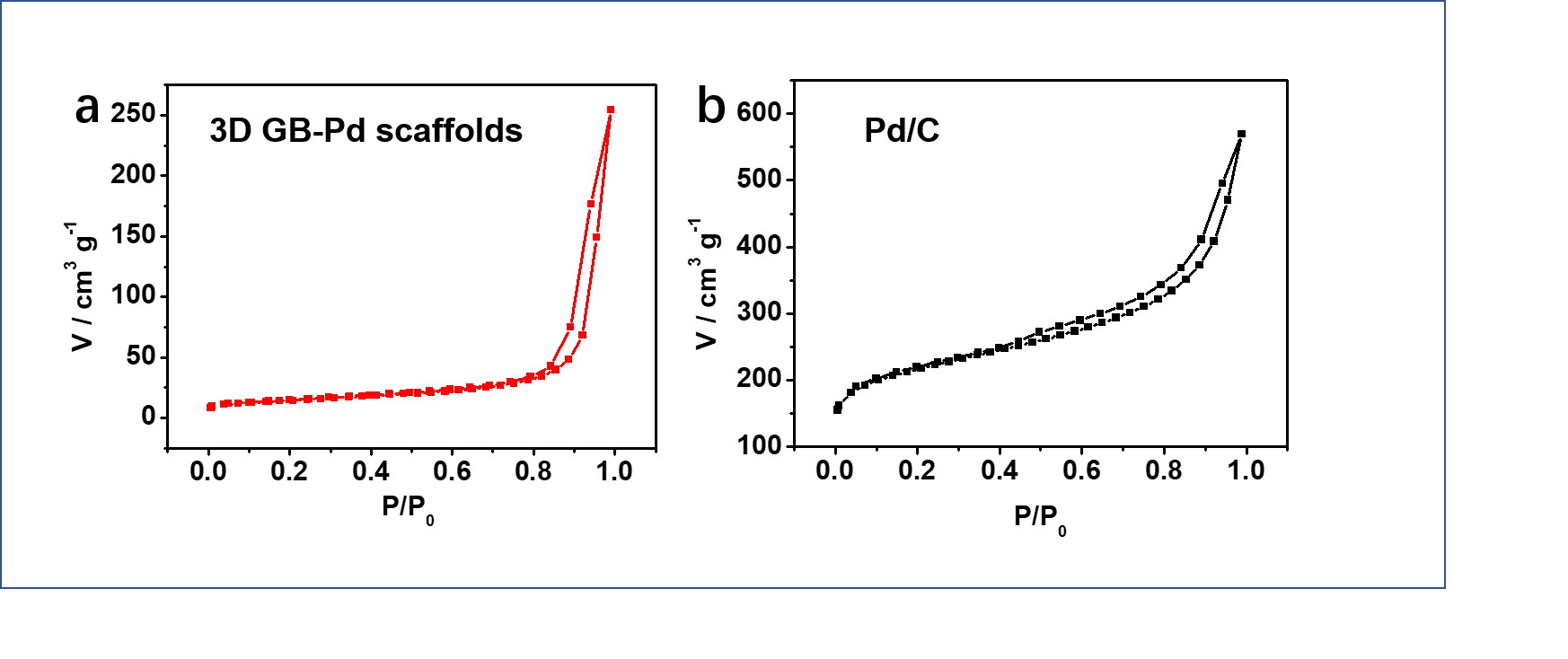


**Figure S21.** (a) Nitrogen adsorption/desorption isotherm of 3D GB-Pd scaffolds. (b) Nitrogen adsorption/desorption isotherm of Pd/C. 3D GB-Pd scaffolds has a Brunauer–Emmett–Teller (BET) surface area of 41.69 m^2^ g^-1^.


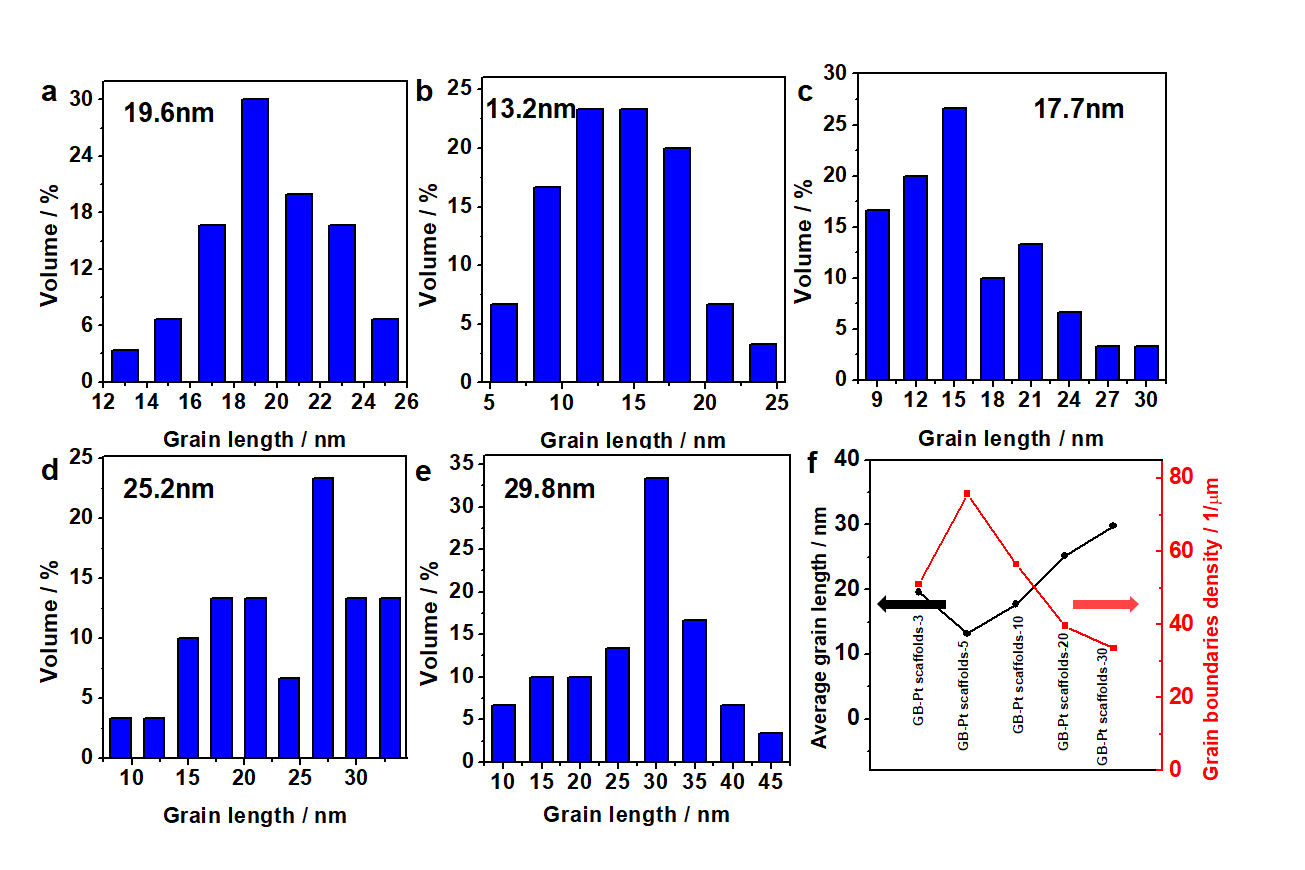


**Figure S22.** Grain length distribution of the 3D GB-Pt scaffolds. (a-e) Grain length distribution of GB-Pt scaffolds-3, GB-Pt scaffolds-5, GB-Pt scaffolds-10, GB-Pt scaffolds-20 and GB-Pt scaffolds-30, respectively. (f) Average grain length and grain boundaries density of the 3D GB-Pt scaffolds.


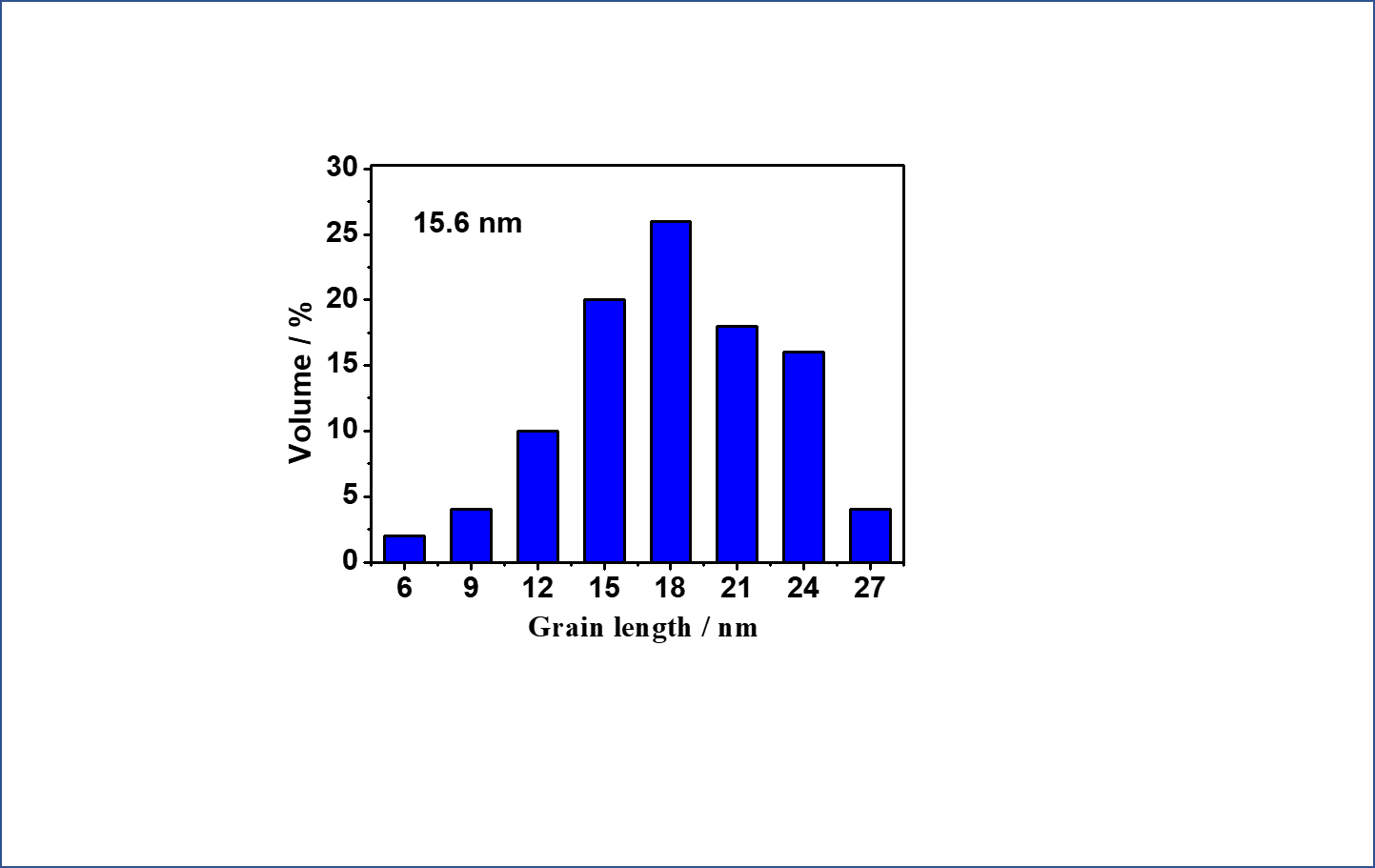


**Figure S23.** Grain length distribution of the 3D GB-Pd scaffolds, showing that 3D GB-Pd scaffolds with an average grain length of 15.6 nm and grain boundaries density of 64.1 1/μm.

**Figure S24.** XRD patterns of the 3D GB-Pt scaffolds.


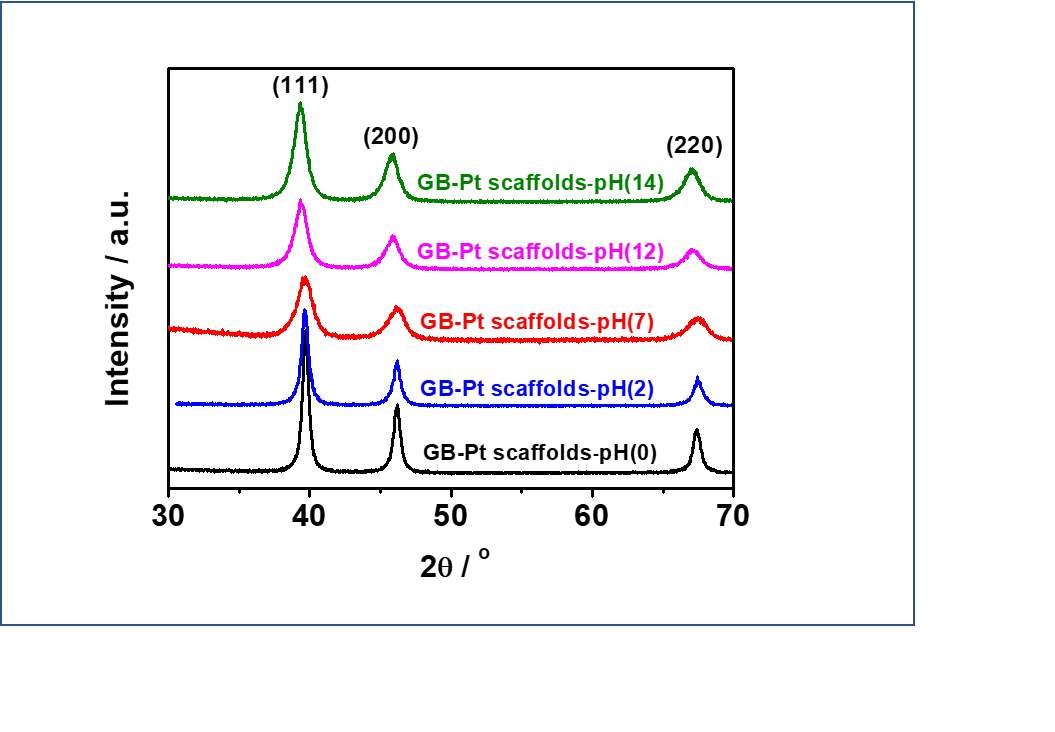


**Figure S25.** XRD patterns of the 3D GB-Pt scaffolds prepared from different pH values of the (NH_4_)_2_PtCl_6_ solution.

**Figure S26.** XPS spectra of the 3D GB-Pt scaffolds.


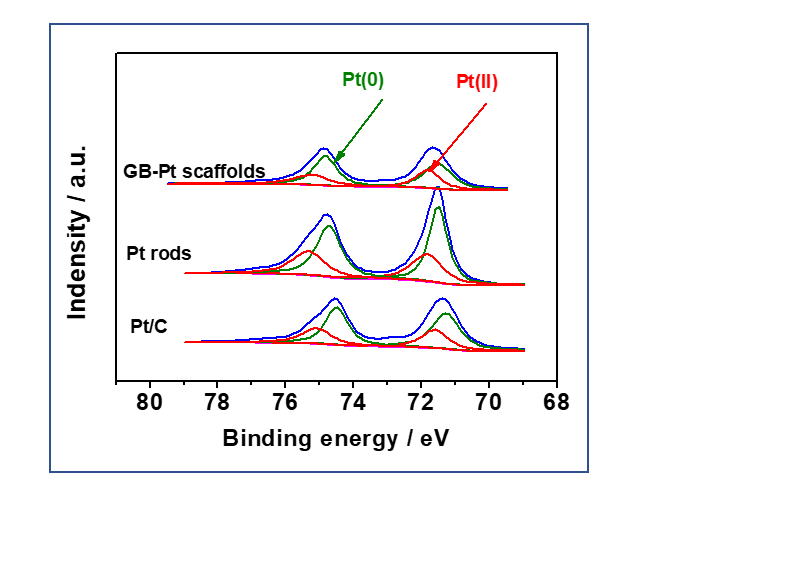


**Figure S27.** Pt 4f spectra of the 3D GB-Pt scaffolds, Pt rods and Pt/C samples. The corresponding high-resolution Pt 4f spectra of the GB-Pt scaffolds shows two separated peaks centered at 71.1 and 74.4 eV, which can be assigned to metallic state Pt (0). Besides Pt (0) signals, additional peaks corresponding to Pt (II) can be observed, suggesting partially oxidation states of Pt.


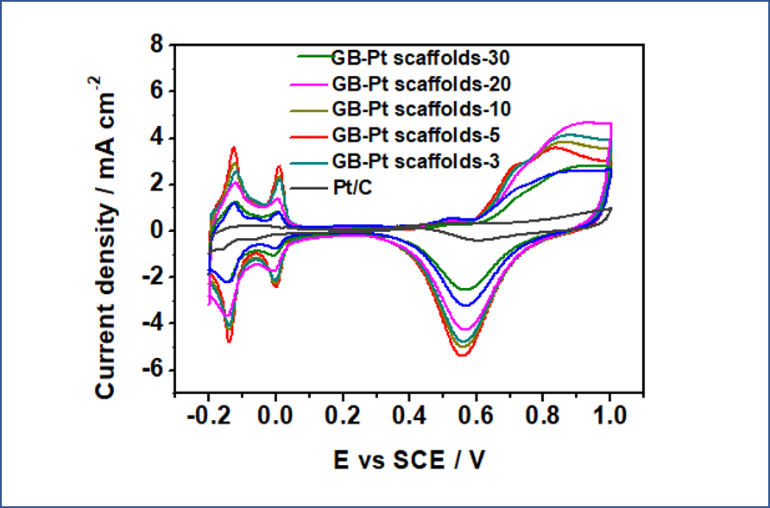


**Figure S28.** Cyclic voltammetry curves of the 3D GB-Pt scaffolds in 0.5M H_2_SO_4_ electrolyte at a sweep rate of 10 mV s^-1^.


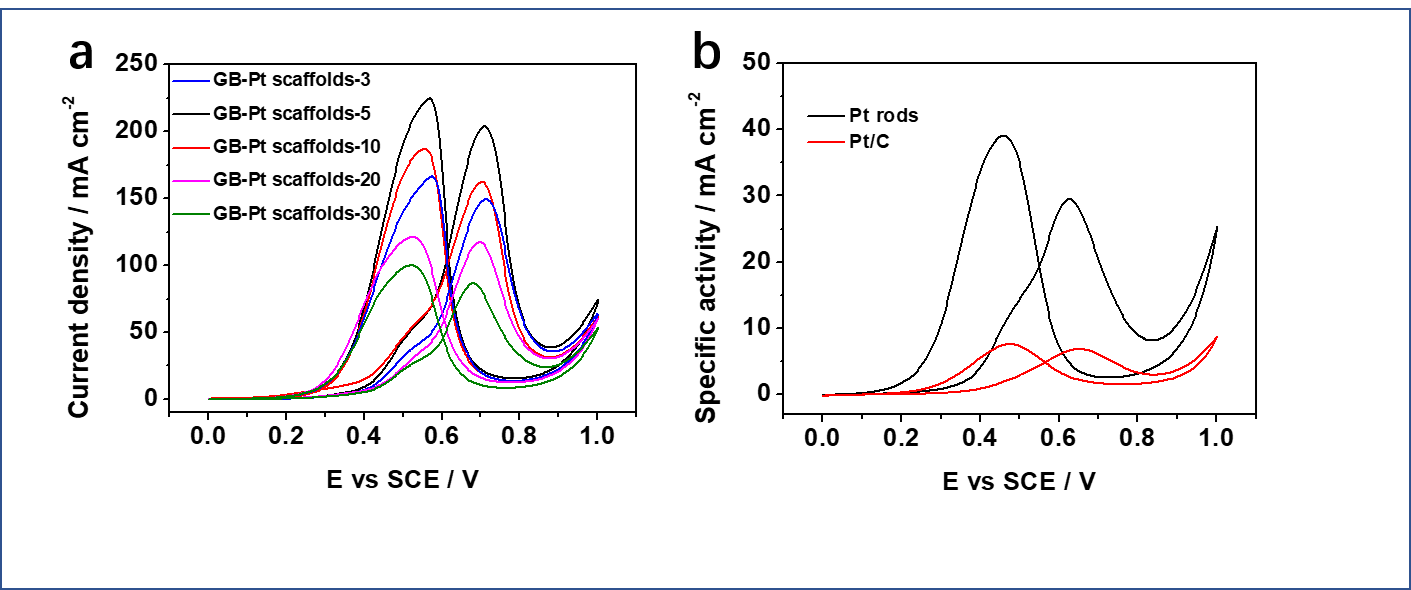


**Figure S29.** Electrocatalytic MOR properties of (a) 3D GB-Pt scaffolds, (b) Pt rods and Pt/C catalysts.


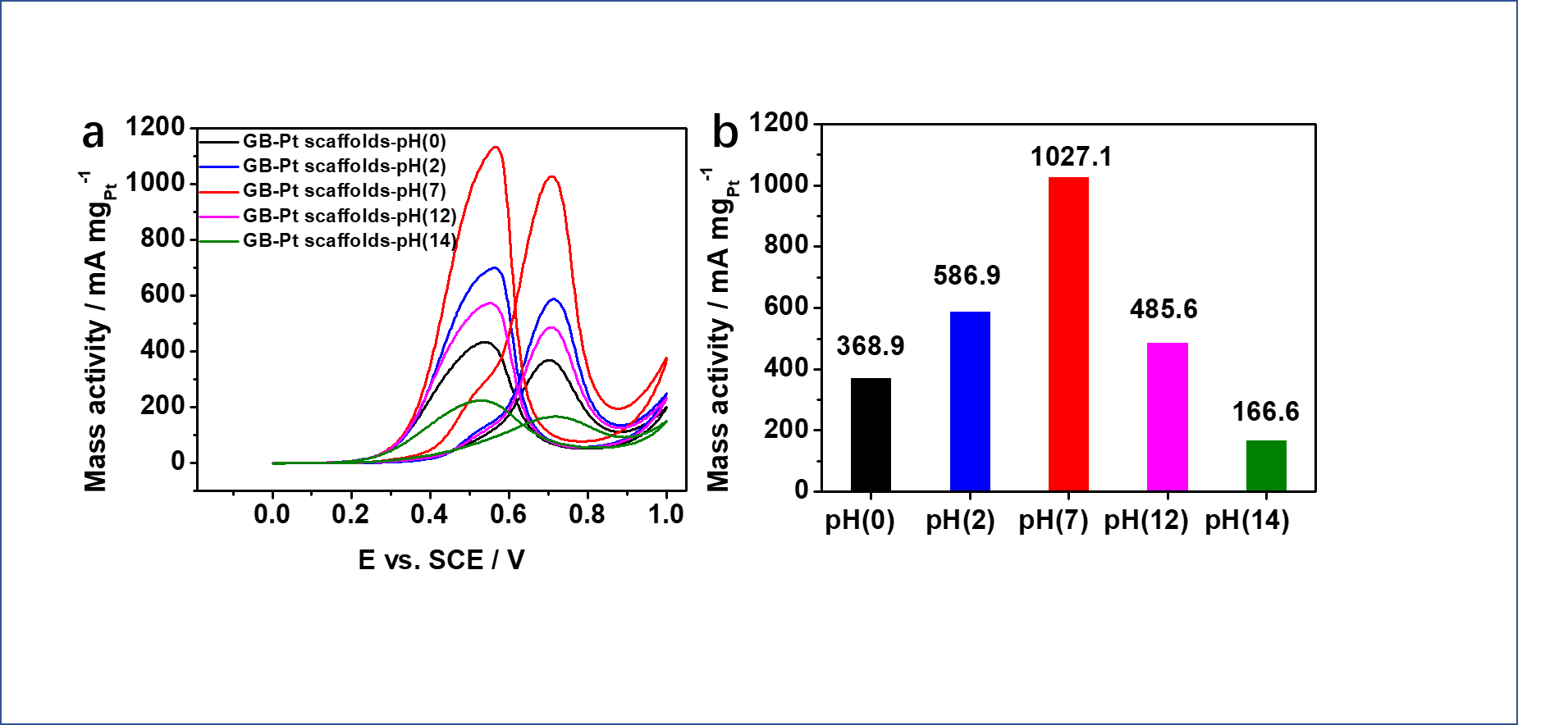


**Figure S30.** Electrocatalytic MOR properties of the 3D GB-Pt scaffolds-pH(0),(2),(7),(12),(14). (a) CVs tested in 0.5M H_2_SO_4_ and 1M methanol electrolyte with 20 mV s^-1^. (b) Comparative Pt mass activity of these catalysts.

**Figure S31.** CO tolerance of 3D GB-Pt scaffolds and Pt/C catalysts measurement by stripping voltammetry of adsorbed CO, indicating that 3D GB-Pt scaffolds have enhanced anti-poisoning (CO) property.


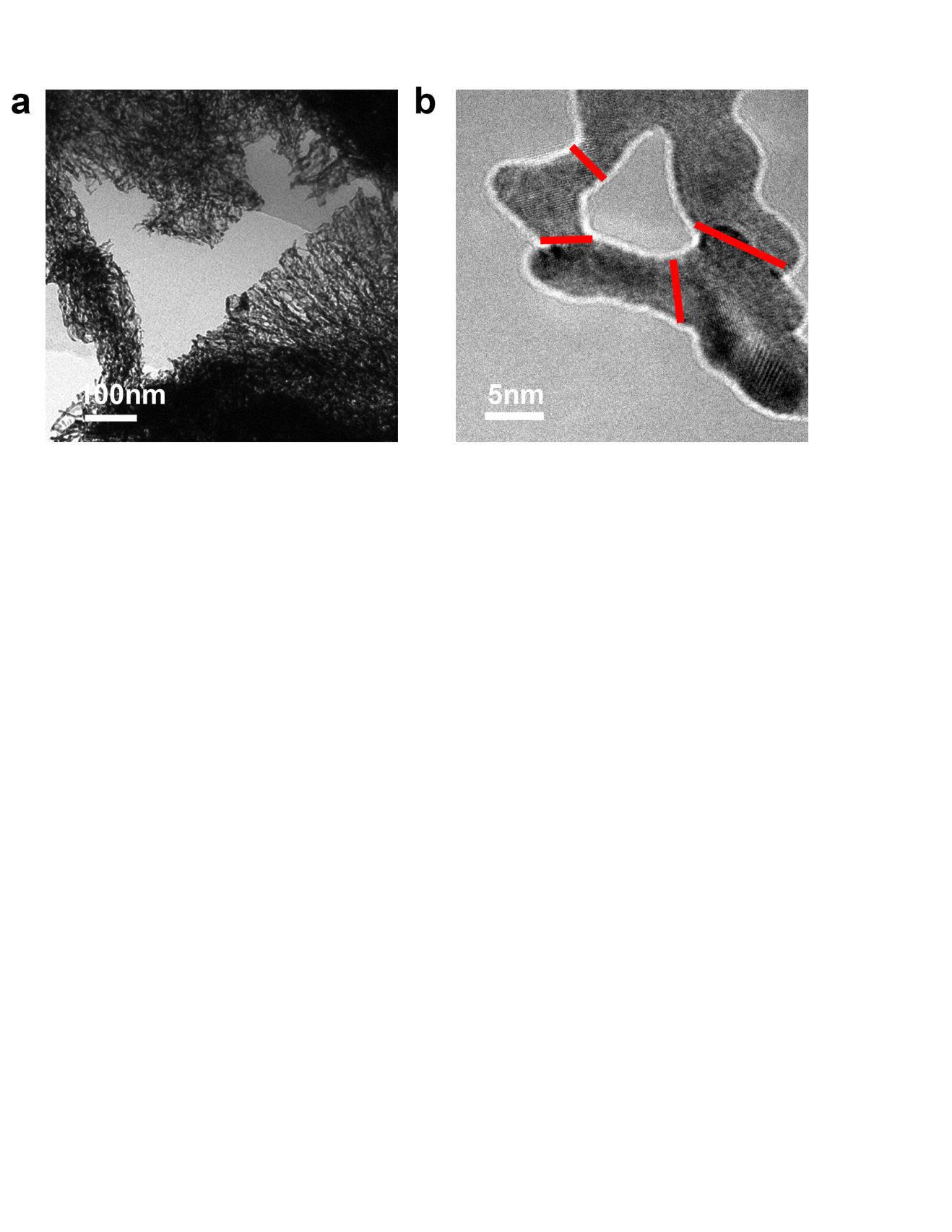


**Figure S32.** (a) TEM and (b) HRTEM images of 3D GB-Pt scaffolds after 5000 cycles.


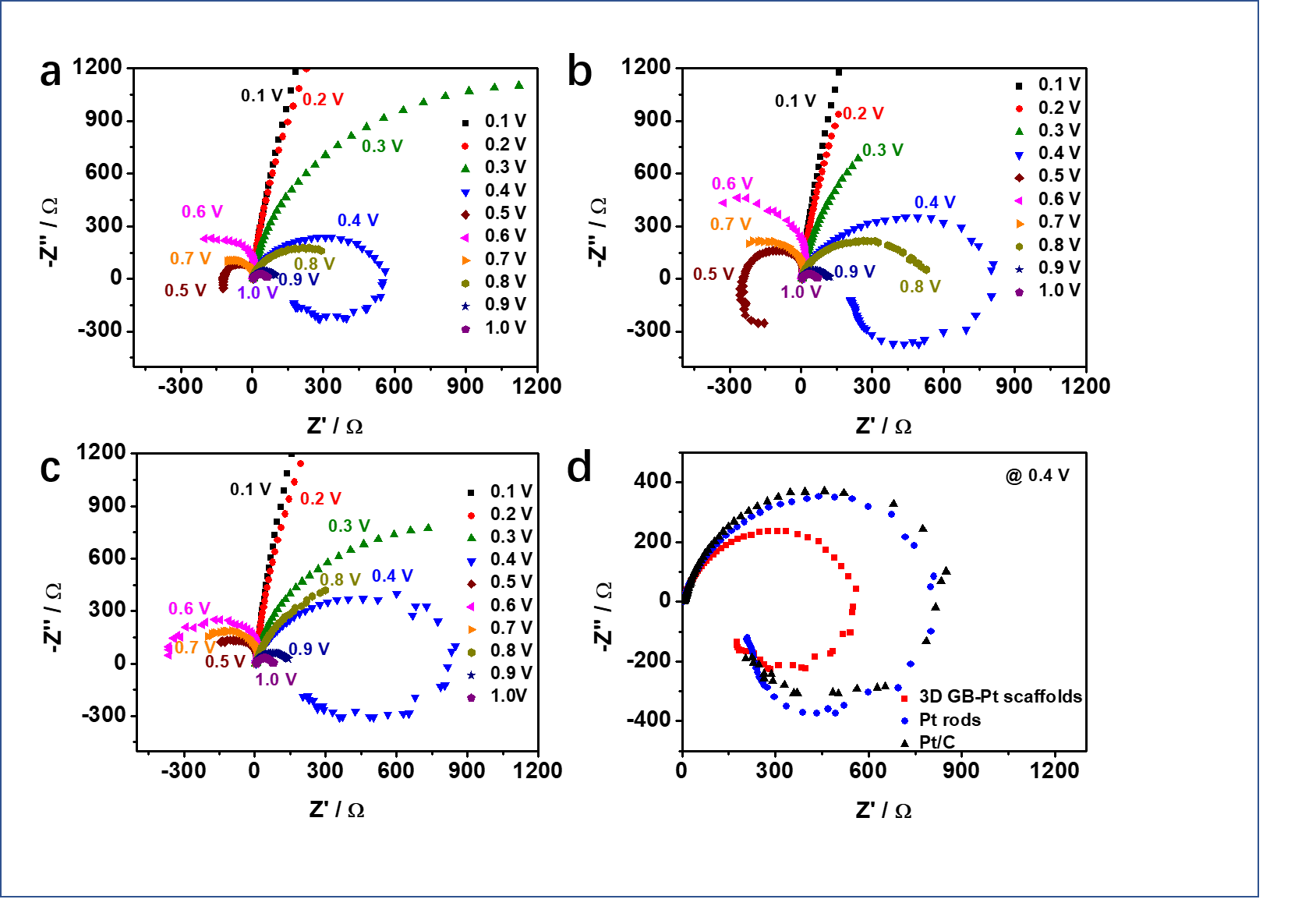


**Figure S33.** Nyquist plots for the 3D GB-Pt scaffolds, Pt rods and Pt/C electrocatalysts. (a-c) Nyquist plots for (a) 3D GB-Pt scaffolds, (b) Pt rods and (c) Pt/C in electrochemical methanol oxidation at different potentials. (d) Nyquist plots of 3D GB-Pt scaffolds, Pt rods and Pt/C electrocatalysts for methanol oxidation at 0.4 V. The Nyquist plot for all the electrocatalysts at different potentials are displayed in Figure S33, and typical methanol oxidation behaviors catalyzed by Pt catalyst are observed. Taking 3D GB-Pt scaffolds as an example, as shown in Figure S33a, the nearly straight lines below 0.3 V mean no methanol oxidation occurred below 0.3V. With increasing applied potential to 0.4 V, a positive loop in the first quadrant was achieved and followed by a low frequency loop in the fourth quadrant. These results confirm that methanol is first dehydrogenated to form adsorbed CO species (CO_ads_) on the surface of Pt, and then oxidatively removed. With potential further increased to 0.5 – 0.7 V, the Nyquist plots mainly located in the second and third quadrants, which indicated that the rate-determining step of methanol oxidation changes from methanol dehydrogenation to oxidation oxidative removal of CO_ads_. At 0.8 – 1.0 V, the shape of the Nyquist plots moves back to the first and forth quadrants, since the adsorption of OH^-^ is too strong at high potentials, which greatly decreases the rate of methanol oxidation.


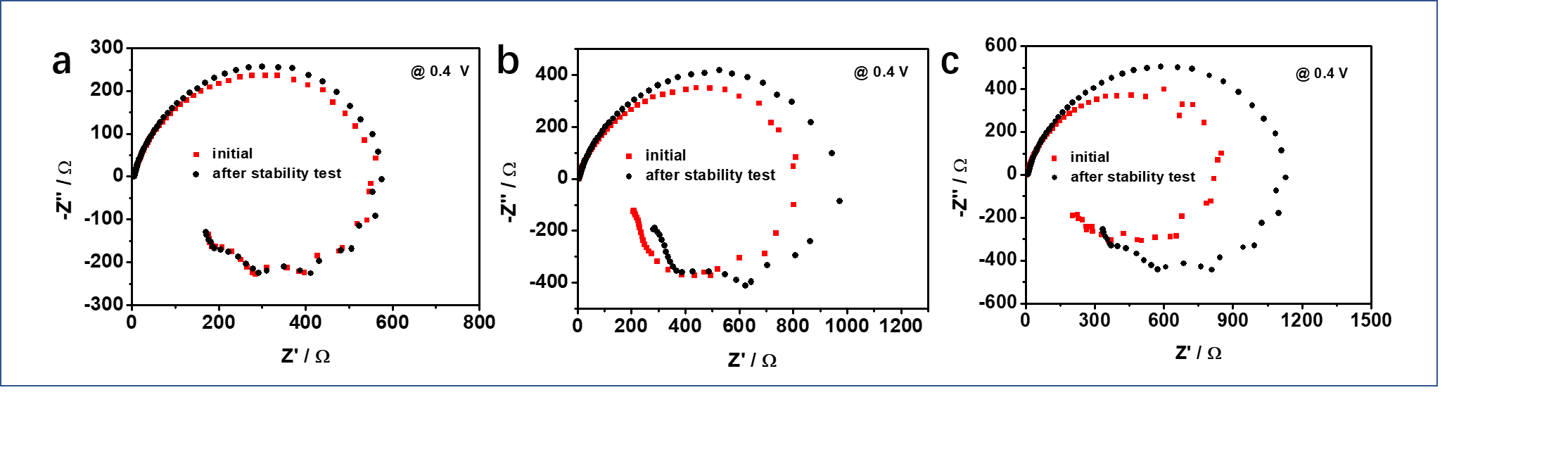


**Figure S34.** (a-c) Nyquist plots of 3D GB-Pt scaffolds, Pt rods and Pt/C electrocatalysts for methanol oxidation before and after stability test. The Nyquist plots of 3D GB-Pt scaffolds show no obvious changes of the diameter before and after stability test. In sharp contrast, both Pt rods and Pt/C shows much higher semicircle diameters after stability test.


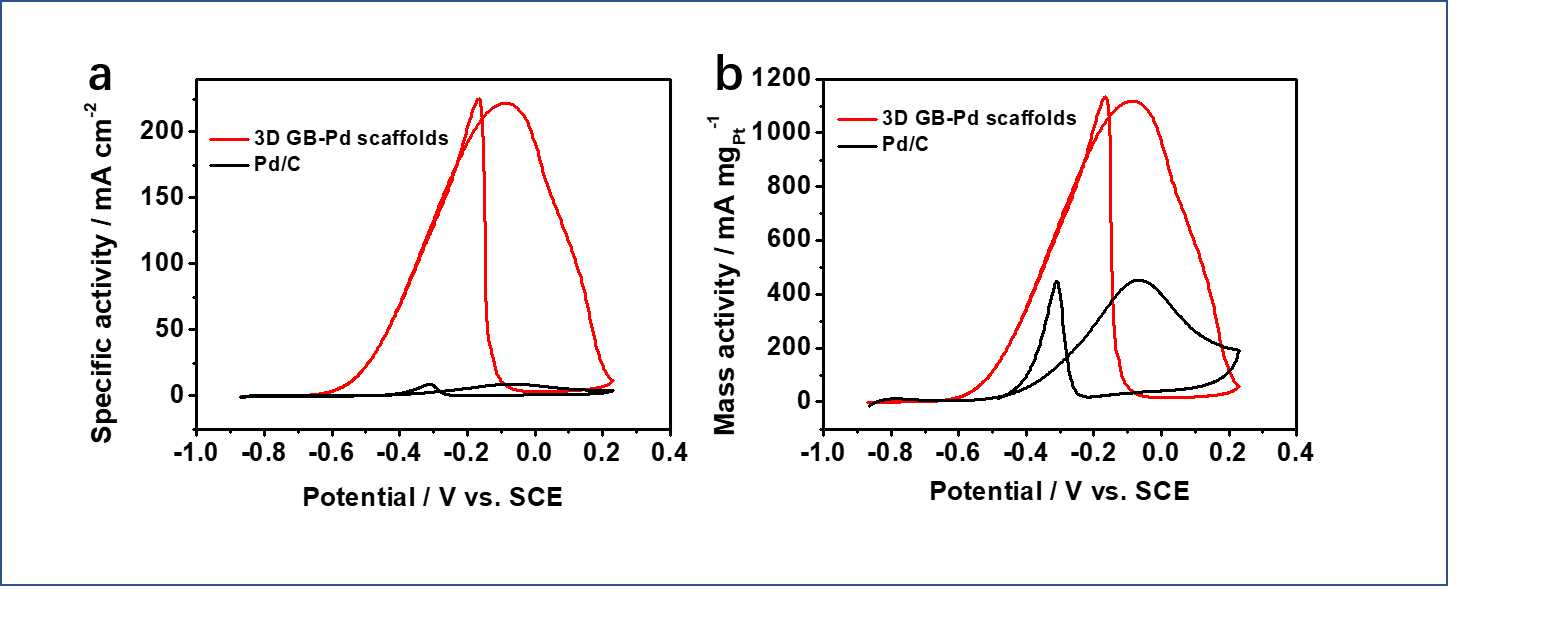


**Figure S35.** (a and b) Comparative Pd specific activity and mass activity of 3D GB-Pd scaffolds and Pd/C (10 wt.%) in 1 M KOH and 1 M methanol electrolyte.


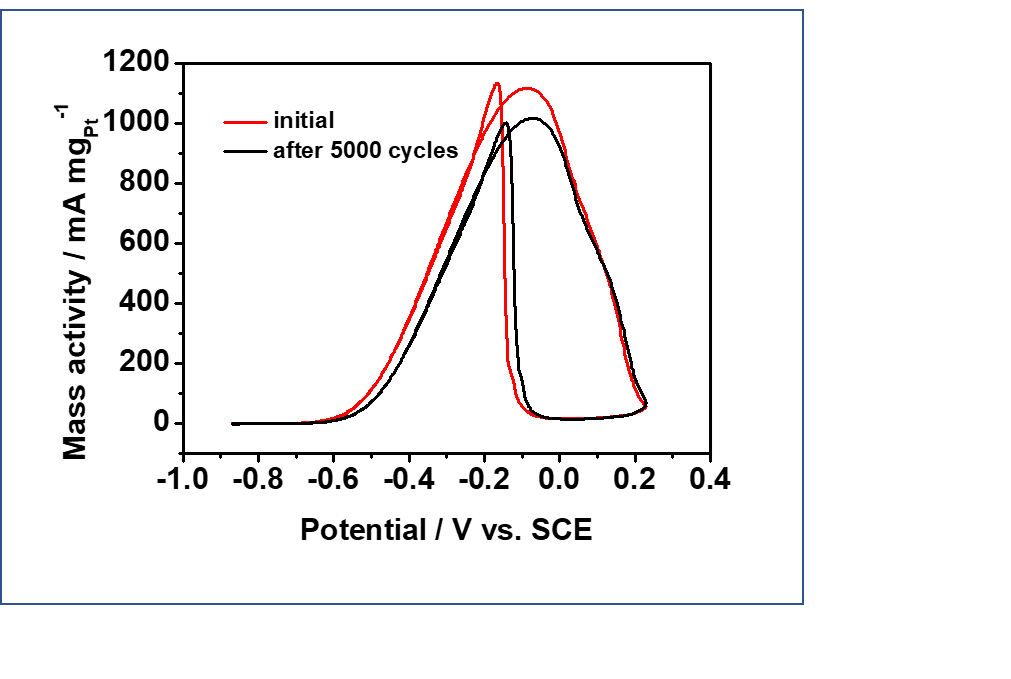


**Figure S36.** CVs of 3D GB-Pd scaffolds before and after 5000 cycles in 1 M KOH and 1 M methanol electrolyte.

**Table S1.** Electrochemical surface area (ECSA) of 3D GB-Pt scaffolds, Pt rods and commercial Pt/C electrocatalysts.

| Sample | ECSA (m^2^ g_Pt_^-1^) |
| --- | --- |
| 3D GB-Pt scaffolds-3 | 61.5 |
| 3D GB-Pt scaffolds-5 | 74.8 |
| 3D GB-Pt scaffolds-10 | 62.7 |
| 3D GB-Pt scaffolds-20 | 47.7 |
| 3D GB-Pt scaffolds-30 | 27.8 |
| Pt rods | 26.9 |
| commercial Pt/C (10 wt. %) | 35.9 |
